# The phosphoinositide coincidence detector Phafin2 promotes macropinocytosis by coordinating actin organisation at forming macropinosomes

**DOI:** 10.1038/s41467-021-26775-x

**Published:** 2021-11-12

**Authors:** Kay Oliver Schink, Kia Wee Tan, Hélène Spangenberg, Domenica Martorana, Marte Sneeggen, Virginie Stévenin, Jost Enninga, Coen Campsteijn, Camilla Raiborg, Harald Stenmark

**Affiliations:** 1 Centre for Cancer Cell Reprogramming, Faculty of Medicine, University of Oslo, Montebello, N-0379 Oslo, Norway; 2 Department of Molecular Cell Biology, Institute for Cancer Research, Oslo University Hospital, Montebello, 0379 Oslo, Norway; 3 Department of Cell and Chemical Biology, Leiden University Medical Center, Einthovenweg 20, 2333 ZC Leiden, The Netherlands; 4 Institut Pasteur, Dynamics of Host-Pathogen Interactions Unit, 25 Rue du Dr. Roux, Paris, France; 5 Department of Molecular Medicine, Institute of Basic Medical Sciences, University of Oslo, PO Box 1112 Blindern, 0317 Oslo, Norway

**Keywords:** Endocytosis, Endosomes

## Abstract

Uptake of large volumes of extracellular fluid by actin-dependent macropinocytosis has an important role in infection, immunity and cancer development. A key question is how actin assembly and disassembly are coordinated around macropinosomes to allow them to form and subsequently pass through the dense actin network underlying the plasma membrane to move towards the cell center for maturation. Here we show that the PH and FYVE domain protein Phafin2 is recruited transiently to newly-formed macropinosomes by a mechanism that involves coincidence detection of PtdIns3P and PtdIns4P. Phafin2 also interacts with actin via its PH domain, and recruitment of Phafin2 coincides with actin reorganization around nascent macropinosomes. Moreover, forced relocalization of Phafin2 to the plasma membrane causes rearrangement of the subcortical actin cytoskeleton. Depletion of Phafin2 inhibits macropinosome internalization and maturation and prevents KRAS-transformed cancer cells from utilizing extracellular protein as an amino acid source. We conclude that Phafin2 promotes macropinocytosis by controlling timely delamination of actin from nascent macropinosomes for their navigation through the dense subcortical actin network.

## Abstract (web summary)

Macropinocytosis permits the cellular uptake of fluids and nutrients via macropinosomes. Here, the authors show that Phafin2 is required for the formation of macropinosomes and permits their transit through dense actin networks.

## Introduction

Macropinocytosis is an actin-dependent endocytosis mechanism that allows cells to take up extracellular fluids and soluble macromolecules by the formation of membrane ruffles that collapse into large vacuoles^1^. Macropinocytosis evolved early in evolution, probably as an uptake mechanism for nutrients in free-living organisms^2^. In mammalian cells, this mechanism is important in dendritic cells and macrophages which use it to sample body fluids for antigens. Macropinocytosis is exploited by pathogens, which trigger macropinocytosis in order to be taken up by host cells^3^. RAS-transformed cancer cells exhibit high levels of macropinocytosis and exploit this mechanism to take up nutrients from the surrounding medium^4^.

Phosphoinositides are key regulators of macropinocytosis. PtdIns(4,5)P_2_ and PtdIns(3,4,5)P_3_ localize to macropinosome cups, where they can trigger actin rearrangements by activating different actin-regulating pathways^5,6^. PtdIns(3,4,5)P_3_ localizes to the rim of macropinosome cups. After ruffle closure, relatively little is known about the role of phosphoinositides. A phosphatase cascade metabolizes PtdIns(3,4,5)P_3_ via PtdIns(3,4)P_2_ and PtdIns3P to PtdIns^7^. Moreover, dephosphorylation of PtdIns(4,5)P_2_ to PtdIns4P by OCRL has recently been shown to be important for macropinosome closure^8^.

After internalization, macropinosomes follow a similar maturation route as endosomes, sequentially gaining markers of early endosomal, late endosomal, and lysosomal identity^5,9,10^. One of the key questions is how macropinosomes mature immediately after their scission from the plasma membrane and how they gain their endosomal identity. Here, we report a maturation stage of macropinosomes immediately after their scission from the plasma membrane and prior to their acquisition of endocytic markers. Their limiting membrane is densely coated with actin, which is then stripped from the vesicle, allowing the macropinosome to escape the actin cytoskeleton and acquire an endosomal identity. We demonstrate that the PH and FYVE domain-containing protein Phafin2 plays a critical role during this process by a mechanism that involves coincidence detection of phosphoinositide pools and direct control of subcortical actin dynamics.

## Results

### Phafin2 shows biphasic localization to macropinosomes

**Fig. 1:**
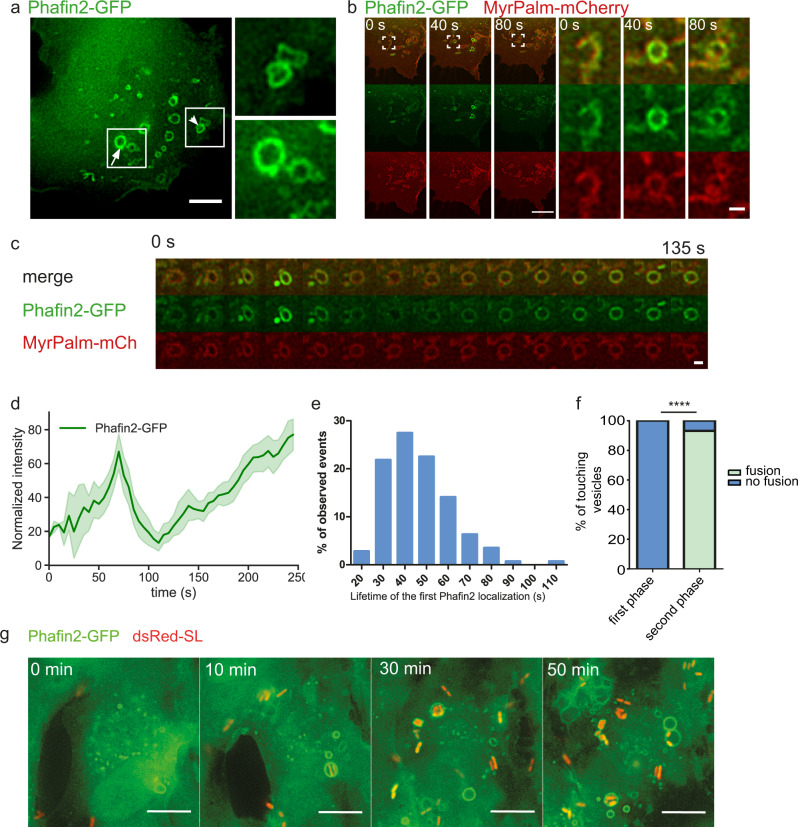
Phafin2 shows biphasic recruitment to nascent and early macropinosomes. **a** Micrograph of RPE1 cells expressing Phafin2 showing labeling on two different vesicle classes - round, endosome-like vesicles within the cell (arrow) and non-uniformly shaped structures close to the cell periphery (arrowhead). Scale bar: 10 µm. Representative image for *n* = 10 cells. **b** Cells co-expressing Phafin2-GFP and MyrPalm-mCherry. Phafin2 transiently localizes to macropinosomes forming from membrane ruffles. Shown are three frames from a time-lapse sequence, spaced 40 s apart. Representative image for *n* = 40 cells. Scale bar: 5 µm, scale bar inset: 1 µm. **c** Sequential images showing Phafin2-GFP dynamics on newly formed macropinosomes; images were acquired every 5 s. Representative image for *n* = 25 macropinosomes. Scale bar: 1 µm. **d** Tracking of individual macropinosomes shows a biphasic Phafin2 localization to macropinosomes. All tracks were temporally aligned to the first peak of Phafin2 fluorescence. *n* = 25 macropinosomes, mean + 95% CI **e** Histogram showing the average life-time of the first Phafin2 localization. *n* = 142 macropinosomes. **f** Macropinosomes labeled with the first Phafin2 localization are fusion incompetent, whereas vesicles readily undergo fusion during the second phase of localization. *n* = 53 macropinosomes. Two-sided Fisher’s exact test, *p* < 0.0001. **g** Phafin2 localizes to *Salmonella*-induced macropinosomes and *Salmonella*-containing vacuoles in RPE1 cells. Representative image for 30 cells. Scale bar: 10 µm. Statistics source data for  **d**, **e**, and **f** are provided in this paper.

We have previously shown that Phafin2 is required for degradation of endocytosed epidermal growth factor receptors^11^. To further elucidate the mechanism of Phafin2 action on endosomes, we performed live-cell-imaging of Phafin2-GFP in immortalized hTERT-RPE1 retinal pigment epithelial cells (RPE1) stably expressing low levels of the fusion protein. Unexpectedly, we observed distinct subcellular localizations for Phafin2. In addition to the previously described endosomal localization, we observed a striking localization to large vesicles with a clearly defined lumen. Moreover, in cells with a high number of membrane ruffles, we observed bright, short-lived bursts of Phafin2 at vesicles in close proximity to the plasma membrane (Fig. 1a, Supplementary Movie S1). Imaging of Phafin2-GFP together with a plasma membrane marker (MyrPalm-mCherry)^12^ revealed that this transient Phafin2 localization occurred at newly formed macropinosomes immediately after the formation of new vesicles from cup-shaped membrane ruffles (Fig. 1b, c, Supplementary Movie S1).

Tracking of individual vesicles showed two distinct phases of Phafin2 localization to the same macropinosome, a short-lived (~40 s), transient localization to structures close to the plasma membrane and a second, long-lasting localization to large vacuoles (Fig. 1d, e). The first localization typically occurred in a single pulse, whereas the second localization was characterized by a gradual increase in fluorescence over several minutes. During the first Phafin2 localization, macropinosomes were irregularly shaped and often appeared to be squeezed and subjected to external forces, whereas the second localization occurred on perfectly round macropinosomes (Fig. 1a). While tracking the fate of individual macropinosomes during the first phase of Phafin2 localization, we noted that these were unable to undergo homotypic fusion, even if they were in extensive contact with neighboring vesicles. In comparison, once they reached the second phase of Phafin2 localization, they readily fused if they were in contact with other macropinosomes (Fig. 1f). Taken together, this indicates that the two phases of Phafin2 localization represent two distinct steps of macropinosome maturation.

To test if Phafin2 localization to forming macropinosomes is a general process which occurs in several biological systems, we monitored Phafin2 localization also in HeLa and HT1080 cells transfected with Phafin2-GFP. In both cell types, Phafin2 showed biphasic localization to forming macropinosomes (Supplementary Fig. S1a, b). Many intracellular pathogens exploit macropinosomes to invade host cells or to establish their replicative niche^13,14^. Following *Salmonella* infection of RPE1 and HeLa cells by time-lapse microscopy, we found that Phafin2 labeled both *Salmonella*-induced macropinosomes and *Salmonella*-containing vacuoles (Fig. 1g, Supplementary Fig. S1c).

### Phafin2 labels macropinosomes prior to recruitment of canonical early endocytic markers

**Fig. 2:**
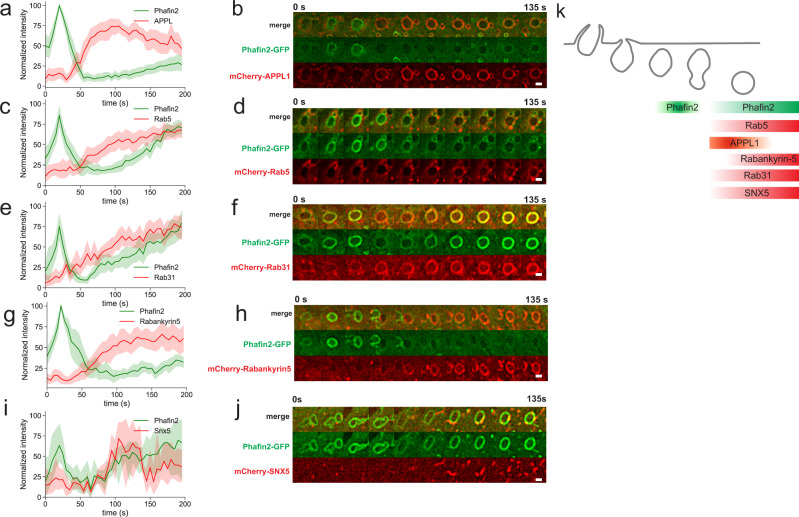
Mapping Phafin2 dynamics in the endocytic pathway. **a** Phafin2 is recruited to forming macropinosomes prior to APPL1. Forming macropinosomes acquire a brief burst of Phafin2. Following this burst, Phafin2 is completely lost from these vesicles, and APPL1 is recruited. During further maturation, APPL1 is gradually replaced by a second, slower recruitment of Phafin2. *n* = 15 macropinosomes; mean + 95% CI. **b** Sequential images showing Phafin2 and APPL1 dynamics on a macropinosome. Representative image for *n* = 15 macropinosomes. Scale bar: 1 µm. **c** Forming macropinosomes acquire a burst of Phafin2, followed by recruitment of RAB5. *n* = 14 macropinosomes, mean + 95% CI. Phafin2 shows a second gradual recruitment. **d** Sequential images showing Phafin2 and RAB5 dynamics on a macropinosome. Representative image for *n* = 14 macropinosomes. Scale bar: 1 µm. **e** RAB31 is recruited after the initial Phafin2 recruitment. *n* = 10 macropinosomes, mean + 95% CI. **f** Sequential images showing Phafin2 and RAB31 dynamics on a macropinosome. Representative image for *n* = 10 macropinosomes. Scale bar: 1 µm. **g** Forming macropinosomes acquire a burst of Phafin2, followed by recruitment of Rabankyrin-5. *n* = 12 macropinosomes, mean + 95% CI. Macropinosome maturation is accompanied by a second, gradual recruitment of Phafin2. **h** Sequential images showing Phafin2 and Rabankyrin-5 dynamics on a macropinosome. Representative image for *n* = 12 macropinosomes. Scale bar: 1 µm. **i** SNX5 is recruited after the initial Phafin2 recruitment. *n* = 4 macropinosomes, mean + 95% CI. **j** Sequential images showing Phafin2 and SNX5 dynamics on a macropinosome. Note that SNX5 localizes to tubules. Representative image for *n* = 4 macropinosomes. Scale bar: 1 µm. **k** Schematic overview of the observed recruitment dynamics. Statistics source data for **a**, **c**, **e**, **g**, **i**, and **k** are provided in this paper.

To characterize the spatiotemporal localization of Phafin2, we imaged the Phafin2-GFP stable cell line with transiently transfected markers of early endosomes and macropinosomes and assessed their recruitment dynamics relative to the first Phafin2 pulse. The early endocytic adapter protein APPL1 (Fig. 2a, b, Supplementary Movie S2)^15^ localizes to newly formed macropinosomes prior to their acquisition of EEA1^9^. APPL1 was recruited after the first Phafin2 pulse had occurred, but prior to the second phase of Phafin2 recruitment. The small GTPases RAB5 (Fig. 2c, d) and RAB31 (Fig. 2e, f)^16^, the RAB5/PtdIns3P effector Rabankyrin-5 (Fig. 2g, h)^10^, and the sorting nexin SNX5 (Fig. 2i, j) also arrived after the first pulse of Phafin2, in parallel with the second gradual localization of Phafin2 to the macropinosome. Snx5 was, in contrast to the other markers, concentrated in subdomains and tubules emanating from macropinosomes. Phafin2 did not localize to clathrin-coated pits by TIRF microscopy, to caveolin-positive structures, or to Endophilin-positive structures (Supplementary Fig. S1d,e,f). These data suggest that Phafin2 is initially also recruited to an immediate-early stage of newly formed macropinosomes that is distinct from and earlier than the better-characterized RAB5-positive maturation step (Fig. 2k).

### Phafin2 localization is dependent on its PH and FYVE domains

**Fig. 3:**
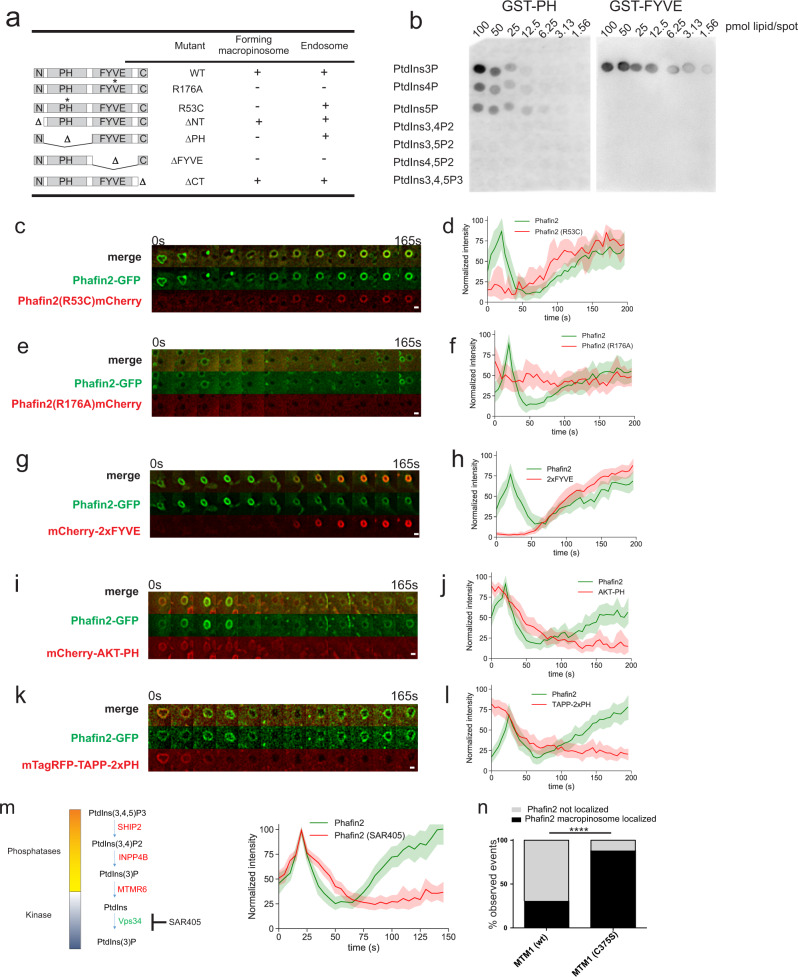
Mutational analysis of Phafin2. **a** Mutational analysis of Phafin2. GFP fusions of Phafin2 truncation and point mutants were tested for their subcellular localization. **b** Protein-lipid binding assay of Phafin2 PH and FYVE domains. **c** Sequential images of a macropinosome showing recruitment dynamics of Phafin2 (WT) and Phafin2 with a defective PH domain (Phafin2(R53C)). Representative image for *n* = 6 macropinosomes. Scale bar: 1 µm. **d** Localization dynamics of Phafin2 and Phafin2 with a defective PH domain (R53C). *n* = 6 macropinosomes, mean + 95% CI. Phafin2 R53C is unable to localize to newly formed macropinosomes, whereas the second, endosomal localization is unaffected. **e** Sequential images of a macropinosome showing recruitment dynamics of Phafin2 (WT) and Phafin2 with a defective FYVE domain (Phafin2 (R176A)). Representative image for *n* = 15 macropinosomes. Scale bar: 1 µm. **f** Localization dynamics of Phafin2 and Phafin2 with a defective FYVE domain (R176A). *n* = 15 macropinosomes, mean + 95% CI. Mutation of the FYVE domain abolishes all membrane localization. **g** Sequential images showing Phafin2 and the PtdIns3P probe mCherry-2xFYVE on a macropinosome. Representative image for *n* = 20 macropinosomes. Scale bar: 1 µm. **h** Localization dynamics of Phafin2 in relation to the PtdIns3P probe mCherry-2xFYVE. *n* = 20 macropinosomes, mean + 95% CI. **i** Sequential images showing Phafin2 and the PtdIns(3,4,5)P_3_ probe AKT-PH on a macropinosome. Representative image for *n* = 19 macropinosomes. Scale bar: 1 µm. **j** Localization dynamics of Phafin2 in relation to the PtdIns(3,4,5)P_3_ probe AKT-PH. *n* = 19 macropinosomes, mean + 95% CI. **k** Sequential images showing Phafin2 and the PtdIns(3,4)P_2_ probe TAPP-2xPH on a macropinosome. Representative image for *n* = 20 macropinosomes. Scale bar: 1 µm. **l** Localization dynamics of Phafin2 in relation to the PtdIns(3,4)P_2_ probe TAPP-2xPH. *n* = 20 macropinosomes, mean + 95% CI. **m** Treatment of Phafin2-GFP expressing cells with the VPS34 inhibitor SAR-405 does not affect the first localization, but abolishes the second localization phase (*n* = 51 macropinosomes each, mean + 95% CI). **n** Overexpression of the PI3 phosphatase MTM1, but not of the catalytically inactive MTM1 (C375S) displaces Phafin2 from macropinosomes (*n* = 75 (WT) and 69 (C375S) cells). Two-sided Fisher’s exact test, *p* < 0.0001. Statistics source data for **c**, **e**, **i**, **k**, and **m** are provided in this paper.

To address how these two different maturation steps, nascent and early macropinosomes, are recognized by Phafin2, we generated a series of GFP-tagged truncation mutants lacking different subdomains and assayed their localization (Fig. 3a). The FYVE domain was required for localization of Phafin2 to both nascent and early macropinosomes. In contrast, the PH domain was critical for localization to the first phase on macropinosomes, but was not required for localization to the second, endosomal phase (Fig. 3a).

We assessed the lipid-binding specificities of the GST-tagged PH and FYVE domains using protein–lipid overlay assays. The PH domain bound to PtdIns3P, PtdIns4P, and PtdIns5P (Fig. 3b), with a slight preference for PtdIns3P, whereas the FYVE domain was highly selective for PtdIns3P^17^ (Fig. 3b). We did not observe binding to any other phosphoinositide species. We generated Phafin2 constructs bearing point mutations that abolish lipid-binding activity in the PH (R53C)^18^ and FYVE (R176A)^19^ domains and assessed their localization behavior. Phafin2(R53C) was still recruited to endosomes and macropinosomes, but the first transient localization to newly formed macropinosomes (Fig. 3c, d, Supplementary Movie S3) was abolished, while Phafin2(R176A) completely lost all membrane association (Fig. 3e, f, Supplementary Movie S4). Thus, PtdIns3P binding by the FYVE domain of Phafin2 is needed for general membrane association, while the first transient localization of Phafin2 also requires lipid-binding by the PH domain.

Surprisingly, despite the selectivity of the Phafin2 FYVE domain for PtdIns3P and strict requirement of its lipid-binding activity for membrane association, we only observed minimal colocalization with an HRS-derived 2xFYVE probe for PtdIns3P^20,21^ (Fig. 3g, h) during the initial Phafin2 pulse. In contrast, 2xFYVE co-localized strongly during the second phase of Phafin2 localization, suggesting that the second wave of Phafin2 localizes to maturing macropinosomes where PtdIns3P is abundant. Both PtdIns(3,4,5)P_3_ and PtdIns(3,4)P_2_ were present at newly formed macropinosomes together with Phafin2 (Fig. 3i–k) and rapidly depleted during the first Phafin2 localization, indicating that lipid substrate for the production of PtdIns3P by this cascade was present.

### Early and late Phafin2 recruitments require PtdIns3P generated by different mechanisms

PtdIns3P can be generated by phosphorylation of PtdIns by class II or III PI3Ks, or by dephosphorylation of 3-phosphorylated phosphoinositide species^7^. To disentangle the contribution of different pathways, we blocked the synthesis of PtdIns3P using SAR-405, an inhibitor of the class III PI3K, VPS34^22^ (Fig. 3m). Treatment with SAR-405 led to a complete displacement of Phafin2 from the endosomal stage (Fig. 3m, Supplementary Movie S5), but preserved the first Phafin2 localization, indicating that the localization of Phafin2 to nascent macropinosomes is VPS34-independent.

To indiscriminately deplete PtdIns3P from all cellular membranes, we overexpressed the PtdIns3P phosphatase MTM1 or a catalytically inactive mutant, MTM1(C375S) as control (Fig. 3n)^23^. Overexpression of MTM1 displaced Phafin2 from both the first and second localization stage, whereas expression of MTM1(C375S) did not affect Phafin2 localization. Likewise, acute release of mitochondrially tethered MTM1 using a reversible dimerization system^24^ resulted in depletion of Phafin2 from forming macropinosomes (Supplementary Movie S6), whereas release of MTM1(C375S) did not affect Phafin2 localization (Supplementary Movie S7). These results suggest that Phafin2 recognizes a VPS34-independent PtdIns3P pool at newly formed macropinosomes and a VPS34-derived endosomal pool at maturing macropinosomes for its biphasic recruitment.

### Phafin2 is a phosphoinositide coincidence sensor

**Fig. 4:**
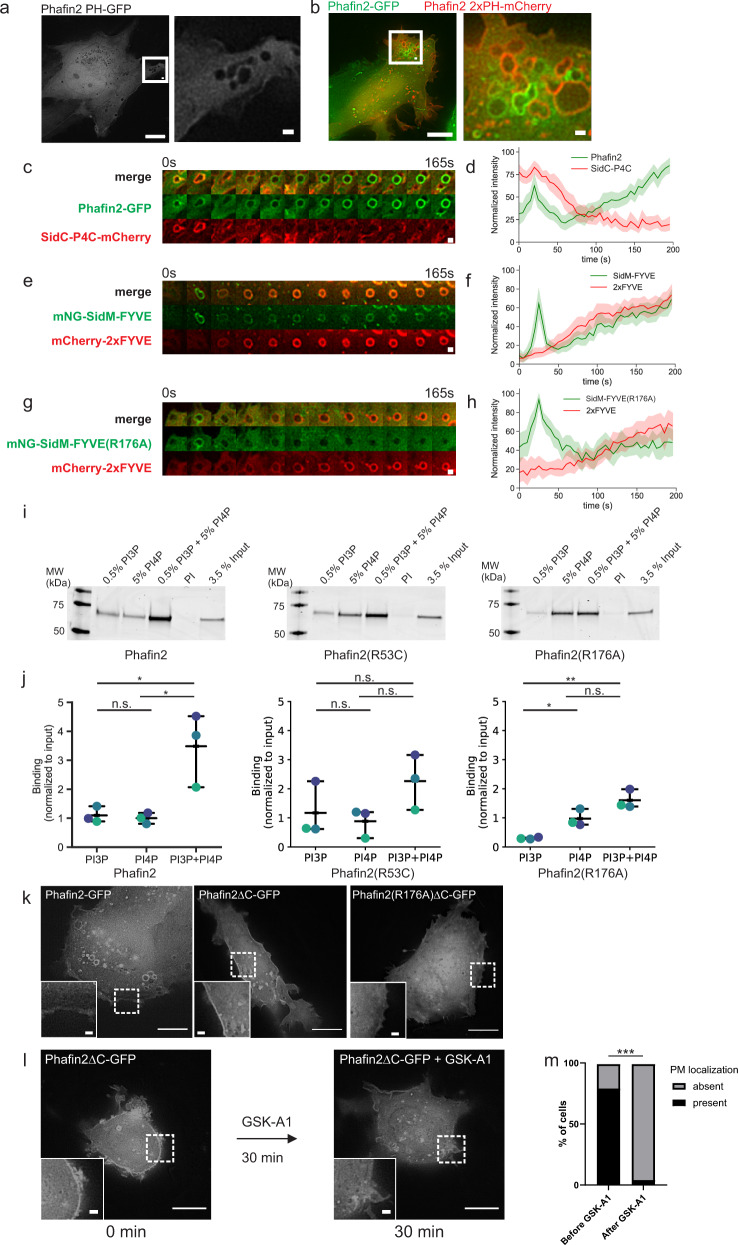
Two independent PtdIns3P pools control the biphasic recruitment of Phafin2. **a** An isolated Phafin2 PH domain does not show membrane localization. Representative image for *n* = 12 cells. Scale bar: 10 µm. **b** A dual Phafin2 PH domain (2xPH) localizes to the plasma membrane and to forming macropinosomes but not to later, endosomal stages. Representative image for *n* = 19 cells. Scale bar: 10 µm. **c** Sequential images showing Phafin2 and the PtdIns4P probe SidC-P4C on forming macropinosomes. Representative image for *n* = 25 macropinosomes. Scale bar: 1 µm. **d** Localization dynamics of Phafin2 in relation to the PtdIns4P probe SidC-P4C. *n* = 25 macropinosomes, mean + 95% CI. **e** Sequential images showing the synthetic coincidence sensor SidM-FYVE^(Phafin2)^ and the PtdIns3P probe 2xFYVE on forming macropinosomes. Representative image for *n* = 25 macropinosomes. Scale bar: 1 µm. **f** Localization dynamics of SidM-FYVE^(Phafin2)^ in relation to the PtdIns3P probe 2xFYVE. *n* = 25 macropinosomes, mean + 95% CI. **g** Sequential images showing the synthetic coincidence sensor SidM-FYVE^(Phafin2)^(R176A) and the PtdIns3P probe 2xFYVE on forming macropinosomes. Representative image for *n* = 25 macropinosomes. Scale bar: 1 µm. **h** Localization dynamics of SidM-FYVE^(Phafin2)^ (R176A) in relation to the PtdIns3P probe 2xFYVE. *n* = 25 macropinosomes, mean + 95% CI. **i** Liposome flotation assays showing Phafin2, Phafin2(R53C) and Phafin2(R176A) binding to liposomes. Shown is the liposome fraction. Representative gel from *n* = 3 experiments. **j** Quantification of Phafin2, Phafin2(R53C) and Phafin2(R176A) binding to liposomes. *n* = 3 experiments, mean + 95% CI. One-way ANOVA with Tukey’s post-test. *p* = 0,0114 (WT), n.s. (R53C) and *p* = 0.0024 (R176A). *p < 0.05, **p < 0.01, n.s. not statistically significant. **k** Exemplary image showing the localization of Phafin2-GFP, Phafin2ΔC, Phafin2ΔC(R176A). Representative image for 20 cells each. Scale bar: 10 µm. **l** Treatment of cells expressing Phafin2ΔC before and after treatment with the PI4K inhibitor GSK-A1. Representative image for 81 cells. Scale bar: 10 µm. **m** Quantification of (**l**). Two-sided Fisher’s exact test, *n* = 81 cells, *p* = 0.0001. Statistics source data and detailed statistics for **d**, **f**, **h**, **j** and **m** are provided in this paper.

While our experiments suggested that PtdIns3P is required Phafin2 localization, they did not address how the PH domain contributes to Phafin2 localization. An isolated PH domain was cytosolic (Fig. 4a), suggesting insufficient affinity to membranes. We generated a tandem construct of the Phafin2 PH domain (2xPH), which did localize to newly formed macropinosomes, but, surprisingly, not to early macropinosomes positive for wild-type Phafin2 (Fig. 4b, Supplementary Movie S8). As endosomes and early macropinosomes are abundant in PtdIns3P, we hypothesized that the PH domain of Phafin2 might bind another phosphoinositide on nascent macropinosomes.

In vitro, the Phafin2 PH domain binds PtdIns3P, PtdIns4P, and PtdIns5P. While PtdIns5P is a very rare lipid, PtdIns4P is abundant in the plasma membrane^25^. Moreover, OCRL produces PtdIns4P by dephosphorylation of PtdIns4,5P_2_ during macropinosome closure^8^. The first phase of Phafin2 localization displayed remarkably similar spatiotemporal dynamics as SidC-P4C, a high-affinity probe for PtdIns4P^26^ (Fig. 4c, d). In line with this, a synthetic construct where we replaced the Phafin2 PH domain with the SidM-P4M PtdIns4P binding domain (SidM-FYVE), faithfully recapitulated both the first and second Phafin2 localization (Fig. 4e, f). The same construct with a defective FYVE domain (SidM-FYVE(R176A)) could still localize to nascent macropinosomes, but did not bind to the second phase (Fig. 4g, h). This suggests that forming macropinosomes contain a sizeable pool of PtdIns4P, which could drive the first localization of Phafin2. It is likely that the PH domain of Phafin2 is only a weak PtdIns4P binder, as an isolated PH domain or Phafin2 with a defective FYVE domain is unable to localize to any membrane structures. Therefore, we hypothesized that the first localization of Phafin2 might rely on coincidence sensing of PtdIns3P by the FYVE domain and PtdIns4P by the PH domain.

To characterize the binding properties of Phafin2 towards membranes of differing phosphoinositide compositions, we performed liposome flotation assays. We used liposomes with an endosome-like lipid composition doped with PtdIns3P (0.5%), PtdIns4P (5%), or both (Fig. 4i, j), with PtdIns to balance phospholipid composition. Phafin2 bound to the liposomes containing a single phosphoinositide species but this was strongly enhanced using liposomes containing both PtdIns3P and PtdIns4P. Phafin2(R53C) bound to liposomes with PtdIns3P, and, surprisingly, also bound to PtdIns4P-containing liposomes. However, binding was not enhanced towards liposomes doped with both PtdIns3P and PtdIns4P. Phafin2(R176A) showed strongly reduced binding to PtdIns3P, whereas binding to PtdIns4P was not affected. For this mutant, the enhanced binding to PtdIns3P/PtdIns4P liposomes was impaired (over 3-fold in wild type vs. <1.5-fold for R176A). These in vitro and in vivo results suggest that minor amounts of PtdIns3P are able to strongly enhance recruitment of Phafin2 to membranes containing PtdIns4P, and that the participation of both the PH and FYVE domains is required.

### The C-terminal acidic tail of Phafin2 suppresses spurious membrane recruitment of Phafin2

Apart from the two lipid-binding motifs, Phafin2 contains a highly acidic and negatively charged C-terminal tail, which was proposed to block the PH domain^27^. We generated a Phafin2 construct lacking this domain (Phafin2ΔC), which remained capable of macropinosome localization but also showed an additional localization to the plasma membrane (Fig. 4k). Plasma membrane recruitment of Phafin2ΔC required binding to PtdIns3P, as Phafin2ΔC with a defective FYVE domain (R176A) was cytosolic (Fig. 4k), in line with the inability of an isolated PH domain to cause membrane association. Phafin2ΔC also required PtdIns4P to localize to the plasma membrane, as inhibition of PI4KIIIα (100 nM GSK-A1), the major source of PtdIns4P at the plasma membrane^28^, effectively removed Phafin2ΔC from the plasma membrane (Fig. 4l, m). Taken together, our data indicate that Phafin2 needs to bind to both PtdIns3P and PtdIns4P for effective membrane recruitment if PtdIns3P is not abundant.

### Phafin2 is required for fluid-phase uptake by macropinocytosis

**Fig. 5:**
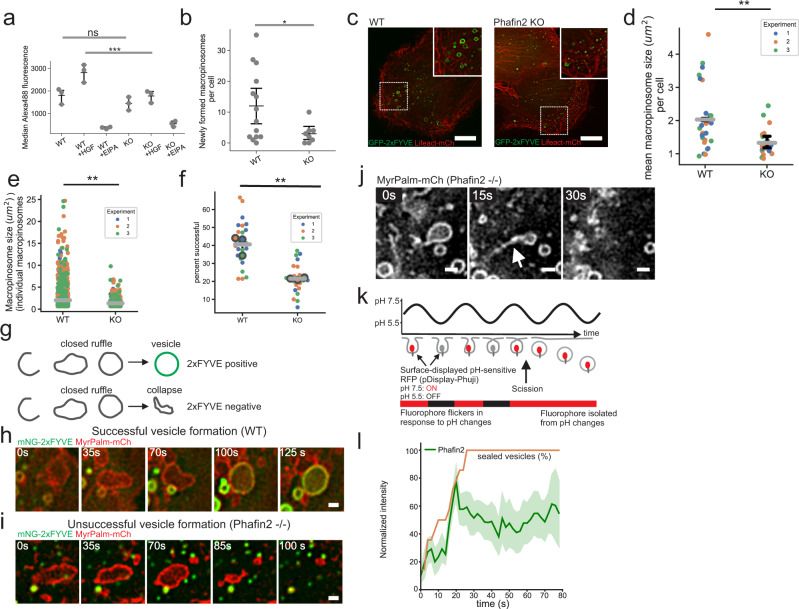
Phafin2 is required for efficient macropinocytosis. **a** Flow cytometry measurement of dextran uptake (10 kDa, Alexa488) in WT and Phafin2 knockout cells. Phafin2 knockout cells show reduced dextran uptake under stimulated conditions. *n* = 3 experiments, mean + 95% CI, ANOVA with Tukey’s post-test, *p* < 0.0001. ***p < 0.001, n.s. not statistically significant. **b** Formation of new macropinosomes (> 1 um in diameter) in Phafin2 WT and KO cells. Macropinosome formation was scored over 20 min of imaging time. *n* = 14 (wt) and 8 (KO) cells, mean + 95% CI, two-sided t-test, *p* = 0.043. *p < 0.05. **c** Exemplary image showing macropinocytosis in WT and Phafin2 knockout cells (single frame extracted from the movies analyzed in (**d**)). Scale bar: 10 µm. **d** Phafin2 knockout cells have smaller macropinosomes than WT cells. Plotted are the mean macropinosome size (> 0.64 µm² area) per individual cell and the mean per experiment + 95% CI. *n* = 3 experiments, two-sided t-test, *p* = 0.0026. **e** Phafin2 knockout cells are unable to form large macropinosomes. Plotted is the size of individual macropinosomes (> 0.64 µm² area) and the mean per experiment + 95% CI. *n* = 3 experiments, two-sided t-test, *p* = 0.0076. **f** Phafin2 knockout cells are less successful in forming macropinosomes. Plotted is the success rate per individual cell and the mean per experiment + 95 % CI. *n* = 3 experiments, two-sided t-test, p = 0.0038. **g** Schematic showing the phenotypes scored in **f**. **h** Sequential images showing successful macropinosome formation in WT cells. Scale bar: 1 µm. Representative images for *n* = 25 cells. **i** Sequential images showing collapsing macropinosomes in Phafin2 knockout cells. Scale bar: 1 µm Representative images for *n* = 24 cells. **j** Sequential images showing macropinosome-associated tubules during collapse of a macropinosome. Scale bar: 1 µm Representative image for 15 cells. **k** Assay measuring the closure of macropinosomes. Cells displaying pH-sensitive pHuji-RFP on their surface were alternatingly perfused with buffers at pH 5.5 and 7.5, and localization of Phafin2 was evaluated in comparison to pHuji. **l** Quantification of Phafin2 localization to forming macropinosomes in relation to vesicle sealing. *n* = 15 macropinosomes, mean + 95% CI. Statistics source data and detailed statistics for **a**, **b**, **d**–**f**, and **l** are provided in this paper.

To assess the functional role of Phafin2 in macropinocytosis, we generated RPE1 cells completely lacking Phafin2 (Supplementary Fig. S2). We measured macropinocytosis using flow cytometry and dextran as a fluid-phase marker. WT cells stimulated with HGF showed robust dextran uptake, whereas Phafin KO strongly reduced dextran uptake (Fig. 5a). This effect was especially pronounced in HGF-treated cells, as unstimulated cells formed only a few and small macropinosomes (Supplementary Fig. S3a, b, c). Depletion of Phafin2 with two different siRNAs resulted in a strong reduction in fluid-phase uptake (Supplementary Fig. S3e, S3f). Phafin2 KO cells also internalized less dextran in a similar microscopy-based assay (Supplementary Fig. 3d). We conclude that Phafin2 is critical for efficient macropinocytosis.

We next used the Phafin2 −/− cell line to follow macropinocytosis by live-cell imaging. Cells were transfected with GFP-2xFYVE to label macropinosomes at the endosomal stage, and with Lifeact-SNAP to label membrane ruffles. In WT cells, we readily observed the formation of multiple large vesicles (Fig. 5b, c, Supplementary Movie S9). In contrast, despite heavy ruffling in Phafin2 KO cells, we only occasionally observed the formation of large vesicles by macropinocytosis (Fig. 5b, c, Supplementary Movie S9).

To monitor the fate of macropinosomes from their formation at the plasma membrane to endosomal stages, we used WT and Phafin2 −/− cell lines stably expressing mNeonGreen(mNG)−2xFYVE and MyrPalm-mCherry and studied these by live imaging and automated vesicle tracking. Tracking of mNG-2xFYVE labeled macropinosomes (>1 µm diameter) showed that macropinosomes in Phafin2 −/− cells were much smaller than in WT cells (Fig. 5d). Moreover, knockout cells completely lacked large macropinosomes (>10 µm^2^) (Fig. 5e). We observed similar effects when we depleted Phafin2 using siRNA. Using Rabankyrin-5 as a macropinosome marker, we noted that cells depleted for Phafin2 showed significantly fewer macropinosomes than control cells, whereas stable expression of siRNA-resistant Phafin2 was able to rescue this defect (Supplementary Fig. S3f–i). These experiments indicate that Phafin2 is required for the formation of normal macropinosomes.

### Phafin2 is required for early steps of macropinosome formation

Phafin2 −/− cells showed robust membrane ruffling, suggesting that these cells should be able to initiate macropinosomes. To address why macropinocytosis was nevertheless inhibited in Phafin2 −/− cells, we tracked the fate of individual macropinosome cups using MyrPalm-mCherry to visualize forming macropinosomes and mNeonGreen-tagged 2xFYVE (mNG-2xFYVE) to indicate the establishment of endosomal identity. In WT cells, ~40 % of macropinosomes successfully gained endosomal identity (Fig. 5f, g, h, i, Supplementary Movie S10). In contrast, in Phafin2 −/− cells, only 20% of forming macropinosomes successfully gained endosomal identity, whereas the rest collapsed and receded to the plasma membrane before mNG-2xFYVE was recruited (Fig. 5f, Supplementary Movie S11). The establishment of endosomal identity, defined by mNG-2xFYVE, was a strong indicator of successful macropinocytosis. The collapse of macropinosomes invariably happened prior to mNG-2xFYVE recruitment, whereas virtually all vesicles that gained mNG-2xFYVE were stable and matured. This suggests that Phafin2 acts at the transition between cup-shaped membrane ruffles and the establishment of endosomal identity.

We observed in rare cases that receding macropinocytic cups appeared to form tubules, which could indicate that they were still connected or had reconnected to the plasma membrane (Fig. 5j, Supplementary Movie S12). To pinpoint the arrival of Phafin2 in relation to sealing of the macropinosome, we performed a pH-based scission assay using a surface-displayed pH-sensitive red fluorescent protein, Phuji^29,30^ (Fig. 5k). Cells were perfused with imaging solutions buffered to pH 5.5 and pH 7.5, alternating every 5 seconds. This caused cycles of quenching and unquenching of Phuji fluorescence on surface-exposed structures but not on sealed vesicles (Supplementary Movie S13; Supplementary Fig. 4a). By measuring Phafin2 intensity and the time point of sealing, we found that sealing directly preceded the recruitment of Phafin2 (Fig. 5l). An analogous assay using the calcium-sensitive protein GCAMP6, which was quenched using EGTA and unquenched by a calcium-containing buffer, also showed that Phafin2 arrived directly after sealing of the vesicle (Supplementary Fig. 4b). This suggests that Phafin2 is not directly involved in the scission of the macropinosome from the plasma membrane, as it is only found on sealed vesicles. Taken together, our findings indicate that Phafin2 is critical for successful macropinosome formation and that it is involved in the step between the closure of the macropinocytic cup and the establishment of endosomal identity.

### The first phase of Phafin2 localization coincides with actin rearrangements around macropinosomes

**Fig. 6:**
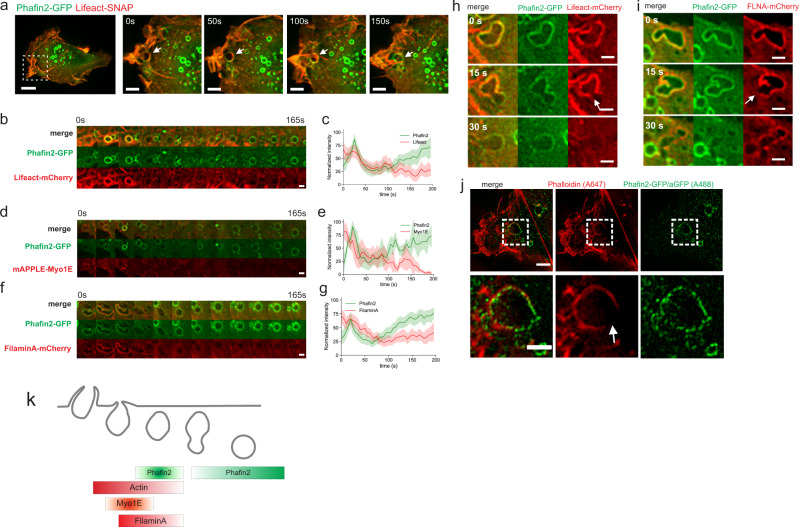
Phafin2 localization coincides with restructuring of the actin cytoskeleton. **a** Light sheet imaging of RPE1 cells expressing Phafin2-GFP and Lifeact-SNAP. Representative image of *n* = 5 cells. Scale bar: 10 µm, inset: 5 µm. **b** Sequential images showing Phafin2 and Lifeact on macropinosomes. Representative image for *n* = 21 macropinosomes. Scale bar: 1 µm. **c** Localization dynamics of Phafin2 and Lifeact at macropinosomes. Lifeact localization shows partial temporal overlap with Phafin2 localization. *n* = 21 macropinosomes, mean + 95% CI. **d** Sequential images showing Phafin2 and Myo1E dynamics on a macropinosome. Representative image for *n* = 6 macropinosomes. Scale bar: 1 µm. **e** Myo1E is recruited earlier than the initial Phafin2 bursts and shows partial temporal overlap during the first phase of Phafin2 recruitment. *n* = 6 macropinosomes, mean + 95% CI. **f** Sequential images of Phafin2 and FilaminA on a macropinosome. Representative images for *n* = 46 macropinosomes. Scale bar: 1 µm. **g** Localization dynamics of Phafin2 and FilaminA on macropinosomes. *n* = 46 macropinosomes, mean + 95% CI. **h** Sequential images showing that actin (visualized by Lifeact) forms a coat around Phafin2-positive macropinosomes. Gaps in the actin coat allow the passage of the Phafin2-labeled vesicle (arrow). Representative image for 18 cells. Scale bar: 1 µm. **i** Sequential images showing that FilaminA forms a coat around Phafin2-positive macropinosomes. Gaps in the FilaminA coat allow the passage of the Phafin2-labeled vesicle (arrow). Representative image for 20 cells. Scale bar: 1 µm. **j** SIM super-resolution image showing a Phafin2-positive macropinosome passing a gap in the actin cytoskeleton (arrow). Representative image for ten cells. Scale bar: 10 µm, inset 2 µm. **k** Schematic overview of the observed recruitment dynamics. Statistics source data for **b**, **d**, and **g** are provided in this paper.

The formation of macropinosomes is tightly linked to actin dynamics^1^. In order to visualize the formation of Phafin2-labeled macropinosomes in relation to actin-driven membrane ruffles, we used high-resolution oblique plane light sheet microscopy (Fig. 6a, Movie S14). We found that the first phase of Phafin2 localization coincides with the major restructuring of the actin cytoskeleton. Actin-coated macropinosomes emerged from the base of membrane ruffles, gained Phafin2, and lost their actin coat. High-resolution live-cell imaging of Phafin2 and the actin marker Lifeact-mCherry showed that newly formed Phafin2-labeled macropinosomes were coated with a dense network of actin filaments (Fig. 6b, c, Supplementary Movie S15). We observed a transient peak of Lifeact-mCherry localization to newly formed macropinosomes, which coincided with Phafin2 recruitment. Actin-associated proteins, such as the actin crosslinking protein FilaminA and the myosin motor protein Myo1E, were present during the first phase of Phafin2 localization. Myo1E—which localizes to macropinosome cups and has been shown to drive phagocytic cup constriction^31–33^, localized directly prior to Phafin2 recruitment (Fig. 6d, e). FilaminA co-localized with Phafin2 on forming macropinosomes and dissociated together with actin (Fig. 6f, g). This indicates that during the first Phafin2 localization, macropinosomes are coated in a dense, highly crosslinked actin network.

Live-cell imaging of Phafin2 and either Lifeact or FilaminA showed that Phafin2 localization of macropinosomes coincided with the removal of this actin network from the macropinosome. During this “uncoating” process, macropinosomes were subject to squeezing forces that pushed them into the inner parts of the cell (Fig. 6h, i, Supplementary Movies S16 and S17). Moreover, we observed gaps in the actin cytoskeleton lining the macropinosomes by both live-cell and 3D SIM imaging (Fig. 6h,i, j). These gaps were visible in both FilaminA and Lifeact expressing cells and coincided with the first Phafin2 localization. Phafin2-positive vesicles were squeezed through these gaps, which allowed the vesicles to leave their actin coat behind and acquire a completely round-vesicle-like shape (Supplementary Movie S16). Directly after vesicles were forced through this gap, they lost the first Phafin2 localization and gained endosomal identity (Fig. 6k).

**Fig. 7:**
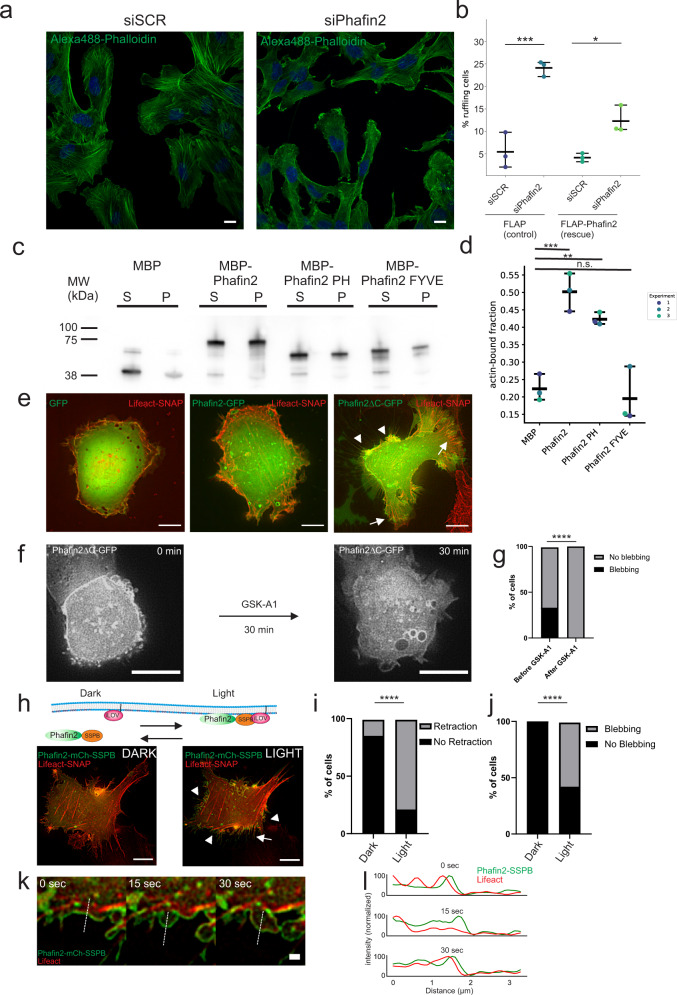
Phafin2 can bind to actin. **a** Confocal images showing the effect of Phafin2 depletion on the actin cytoskeleton. Representative image of the data scored in Fig. 7b. Scale bar: 10 µm. **b** Depletion of Phafin2 results in an increased number of cells with membrane ruffles, which can be rescued by expression of siRNA-resistant Phafin2. Data derived from high content imaging (2038, 1581, 1831, 2055 cells for siSCR, siPhafin2, siSCR(Rescue) and siPhafin2(Rescue) in total. *n* = 3 experiments, mean + 95% CI, two-sided *t*-test, *p* = 0.0017 (Control), *p* = 0.017 (Rescue). **c** Western blot showing in vitro actin-binding assays of MBP-Phafin2 and Phafin2 mutants. Phafin2 and the isolated Phafin2 PH domain co-sediment with actin. MBP or the isolated Phafin2 FYVE domain does not co-sediment. **d** Quantification of the in vitro actin-binding assays. *n* = 3 experiments, mean + 95% CI, ANOVA with Tukey’s post-test, *p* = 0.0009 (MBP vs Phafin2), *p* = 0.0073 (MBP vs. Phafin2 PH), *p* = 0.91 (MBP vs Phafin2 FYVE). **e** Images showing the effect of GFP, Phafin2-GFP, and Phafin2ΔC-GFP expression in RPE1 cells expressing Lifeact-SNAP. Representative image of 30 cells each. Arrowheads indicate cell retractions/ membrane blebs; arrows indicate flat cell protrusions. Scale bar: 10 µm. **f** Time-lapse images of cells expressing Phafin2ΔC before and after incubation with the PI4K inhibitor GSK-A1. Scale bar: 10 µm. **g** Quantification of membrane blebbing after treatment of Phafin2ΔC-expressing cells with the inhibitor GSK-A1. *n* = 81 cells. Two-sided Fisher’s exact test, *p* < 0.0001. **h** Time-lapse images of cells expressing light-dimerizable Phafin2 (Phafin2-mCherry-SSBP and Venus-iLID-CAAX) and Lifeact before and after light-induced plasma membrane recruitment. Arrowheads indicate membrane blebs, arrows cell retraction. Representative image of 25 cells. Scale bar: 10 µm. **i** Quantification of cell retraction after light-induced membrane recruitment of Phafin2. *n* = 28 cells, two-sided Fisher’s exact test, *p* < 0.0001. **j** Quantification of membrane blebbing after light-induced membrane recruitment of Phafin2. *n* = 28 cells, two-sided Fisher’s exact test, *p* < 0.0001. **k** Time series showing a forming and retracting membrane bleb—visualized by membrane-recruited Phafin2-and Lifeact after Phafin2 membrane recruitment. Scale bar: 10 µm. **l** Line plots showing intensities of membrane-recruited Phafin2 and Lifeact over time. Statistics source data for **b**, **d**, **g**, **i**, **j**, and **l** are provided in this paper.

Interestingly, we observed that macropinosomes in cells lacking Phafin2 retained FilaminA around the limiting membrane until they ultimately collapsed (Supplementary Fig. S5a, b, c, Supplementary Movie S18), suggesting that in Phafin2 KO cells, macropinosomes are enclosed in a densely crosslinked actin coat. Depleting FilaminA suppressed the Phafin2 KO phenotype and allowed formation of large macropinosomes in Phafin2 KO cells (Supplementary Fig. S5d, e), suggesting that the Phafin2 KO phenotype might be caused by a failure to remove the actin coat surrounding the macropinosome, which can be remedied by reducing actin crosslinking. These observations pose the question of how Phafin2 and actin act during macropinosome formation. We observed that cells depleted for Phafin2 by siRNA showed a strongly increased number of membrane ruffles, whereas the introduction of a siRNA-resistant allele reduced this effect (Fig. 7a, b). This led us to ask if Phafin2 could influence actin dynamics at forming macropinosomes.

### The Phafin2 PH domain binds directly to F-actin

Some PH domains, such as the PH domain of BTK^34^, can directly bind to F-actin. Based on the close interaction of Phafin2 and actin, we therefore tested if Phafin2 can directly bind to F-actin via its PH domain. We performed in vitro F-actin binding assays—using recombinant His_6_-MBP-fused Phafin2, His_6_-MBP-PH, and His_6_-MBP-FYVE domains, as well as His6-MBP as a control—and measured the F-actin-bound fraction of Phafin2. Both full-length Phafin2 and the Phafin2 PH domain showed robust binding to filamentous actin, whereas the Phafin2 FYVE domain did not show binding above the background (Fig. 7c, d). This indicates that Phafin2 can bind directly to F-actin and that this binding is mediated by the PH domain.

### Phafin2 modulates actin cytoskeleton dynamics

Phafin2 is transiently recruited to forming macropinosomes, and the recruitment coincides with the uncoating of the macropinosome from actin. It is, therefore, tempting to speculate that Phafin2 could locally modulate actin dynamics in close proximity to the plasma membrane. We therefore tested if membrane-associated Phafin2 could modulate actin dynamics. For this, we exploited our observation that Phafin2 lacking the C-terminus (Phafin2ΔC) localized not only to forming macropinosomes but also to the plasma membrane (Fig. 4i–k). First, we tested if Phafin2 requires actin for its recruitment by treating cells expressing Phafin2ΔC with Latrunculin B (Supplementary Fig. S6a, b). This treatment did not affect Phafin2ΔC localization, indicating that Phafin2 localization is primarily driven by phosphoinositide binding and not by actin-binding.

We observed that overexpression of Phafin2ΔC triggered massive changes in cell morphology and the underlying actin cytoskeleton. Cells expressing Phafin2ΔC showed cell retractions and membrane blebs (Fig. 7e, Supplementary Movie S19). Membrane ruffling was largely suppressed; instead, flat membrane protrusions with multiple filopodia were formed (Fig. 7e). Cells expressing high levels of Phafin2ΔC were rounded and showed amoeboid-like cell migration (Supplementary Movie S20).

To test if this is caused by membrane-localized Phafin2, we treated cells expressing Phafin2ΔC with GSK-A1, which effectively removes Phafin2ΔC from the plasma membrane. The addition of GSK-A1 displaced Phafin2ΔCT from the plasma membrane and suppressed the retraction and membrane blebbing (Fig. 7f, g, Supplementary Movie S21), indicating that membrane-localized Phafin2, and not simple overexpression of Phafin2ΔC, triggers these phenotypes.

In order to allow tight temporal control of these phenotypes, we used both rapamycin- and light-inducible dimerization^35,36^ to recruit wild-type Phafin2 to the plasma membrane. Recruitment of Phafin2 to the plasma membrane by either system triggered rapid cell retraction and membrane blebbing (Fig. 7h, i, j, Supplementary Fig. S6d–f, Supplementary Movies S22, S23, and S24). Live-cell imaging of cells expressing Lifeact-SNAP and light-recruitable Phafin2 showed that the membrane in these blebs was first devoid of actin (Fig. 7k,l), suggesting a delamination of the membrane from the actin cytoskeleton. Taken together, our data indicate that Phafin2 is recruited to membranes, where it can bind to actin and locally modulate actin dynamics.

### Phafin2 allows macropinocytosis for nutrient scavenging in cancer cells

**Fig. 8:**
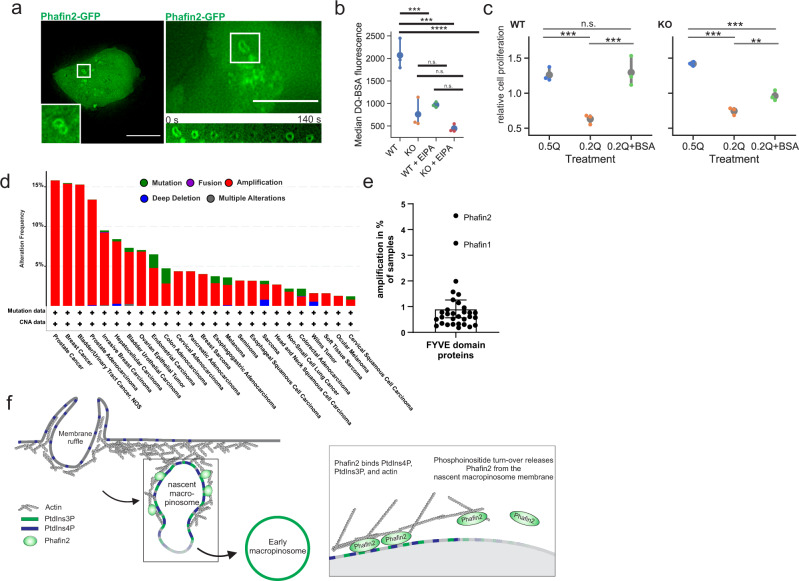
Phafin2 is required for amino acid scavenging by cancer cells. Localization of Phafin2 in MIA-PACA2 cells. **a** Phafin2-GFP labels large vacuoles and shows biphasic localization to macropinosomes in MIA-PACA2 cells. Representative images from 10 cells. Scale bar: 10 µm. **b** Flow cytometry measurement of DQ-BSA by MIA-PACA2 cells. Deletion of Phafin2 strongly reduces BSA uptake. *n* = 3 experiments, mean + 95% CI, ANOVA with Tukey’s post-test, *p* = 0.0002. **c** MIA-PACA2 cells lacking Phafin2 are unable to scavenge extracellular nutrients. WT cells can scavenge extracellular proteins under amino acid limiting conditions. KO cells are unable to utilize extracellular proteins. *n* = 6 experiments, shown are mean + 95% CI, ANOVA with Tukey’s post-test, *p* = 0.0001. **d** Phafin2 is frequently amplified in cancer samples. A screen of the cBioportal cancer database shows frequent amplification of Phafin2 in cancer samples, whereas deletions or mutations are rare. **e** Phafin2 and the related protein Phafin1 are the only frequently amplified FYVE domain proteins. Shown is the frequency of amplification of 31 FYVE proteins in the curated dataset of non-redundant studies in the cBioPortal (representing 48834 non-redundant samples). Mean + /− 95% CI. **f** Model of Phafin2 function on forming macropinosomes. Phafin2 binds to newly formed macropinosomes by coincidence sensing of PtdIns3P and PtdIns4P. By binding to filamentous actin, Phafin2 links the macropinosome membrane to the actin cytoskeleton. Turnover of phosphoinositides leads to a dissociation of Phafin2 from the macropinosome. Statistics source data for **b**, **c**, **e** are provided in this paper.

Finally, we addressed the importance of Phafin2 in a physiological process relying on macropinocytosis, the scavenging of extracellular proteins by KRAS-transformed cancer cells^4,37^. MIA-PACA2 cells—KRAS-transformed pancreatic cancer cells—express Phafin2 (Supplementary Fig. S7), and GFP-tagged Phafin2 labeled large vesicles and showed a biphasic localization pattern in these cells (Fig. 8a). We deleted Phafin2 in MIA-PACA2 cells (Supplementary Fig. S7) and tested their ability to utilize extracellular proteins. Flow cytometry using self-quenched bovine serum albumin (DQ-BSA) showed strongly reduced BSA uptake in Phafin2 −/− cells (Fig. 8b). To test if Phafin2 is required to scavenge extracellular proteins under amino acid limiting conditions, we limited available glutamine and provided BSA as an alternative glutamine source^4^. While WT cells were able to utilize BSA, Phafin2 −/− cells were unable to scavenge extracellular BSA and showed reduced proliferation (Fig. 8c). These results indicate that Phafin2 is required for functional macropinocytosis in cancer cells. Screening the publicly available cBioportal cancer genome database^38,39^ for cancers with alterations in Phafin2 showed that Phafin2 is frequently amplified across multiple cancers, whereas deletions are very rare (Fig. 8d, e). This raises the possibility that moderate overexpression of Phafin2 is advantageous for cancer cells, potentially by supporting improved nutrient scavenging.

## Discussion

In this study, we provide insights into the mechanisms of early macropinocytosis and its regulation by phosphoinositides. We identify Phafin2 as a phosphoinositide-binding regulator of macropinocytosis that marks a previously uncharacterized maturation stage of macropinosomes prior to their acquisition of early endosomal markers. Through binding to actin and causing its timely membrane dissociation, Phafin2 promotes the transition of nascent macropinosomes through the dense subcortical actin network, thereby facilitating macropinocytosis. While Phafin2’s function in macropinocytosis could be beneficial in antimicrobial defense, RAS-transformed cancer cells can co-opt Phafin2-dependent macropinocytosis for proliferation under low-nutrient conditions.

After their formation, macropinosomes undergo drastic changes in their phosphoinositide composition. PtdIns(3,4,5)P_3_ is metabolized via PtdIns(3,4)P_2_ and PtdIns3P to PtdIns^6,7,40,41^, which is then followed by generation of PtdIns3P directly after scission^41^. In parallel, PtdIns4P is generated by dephosphorylation of PtdIns(4,5)P_2 _^8^. So far, only few effectors of these transient lipid pools have been described. Phafin2 is a lipid coincidence sensor that recognizes transient PtdIns4P and PtdIns3P pools by its PH and FYVE domains. This is reminiscent of clathrin-mediated endocytosis, which also has been reported to require coincidence sensors for transient pools of PtdIns3P and PtdIns4P^42,43^.

We find that membrane localization of Phafin2 is tightly controlled. The C-terminus of Phafin2 restricts its localization to forming macropinosomes and acts as a “noise suppressor”, preventing spurious membrane association. Mechanistically, the Phafin2 C-terminus could—in line with a recent proposal^27^—regulate the lipid-binding activity of the PH domain and, by this, prevent membrane binding. Alternatively, the negative charges could suppress Phafin2 binding to low concentration phosphoinositide pools, thus restricting Phafin2 localization to regions with high concentrations of phosphoinositides. Dephosphorylation of PtdIns(4,5)P2 to PtdIns4P by OCRL^8^ or other 5' phosphatases could provide a mechanism to simultaneously reduce negative charges on the membrane and produce PtdIns4P, which would participate in recruitment of Phafin2. Binding of Phafin2 could thus balance between available phosphoinositides and the charge of the plasma membrane, thereby allowing a fine-tuning of Phafin2 localization to membranes.

Phafin2 is critical for the early steps of macropinocytosis, likely by coordinating actin dynamics on newly formed macropinosomes by binding to actin. After scission from the membrane, newly formed vesicles are coated by a dense actin meshwork. Gaps in this network allow the macropinosome to escape its actin coat, thereby enabling maturation and progression into the endolysosomal pathway. While cells lacking Phafin2 still initiate macropinosomes, the formed vesicles remain coated in actin and frequently collapse and likely re-fuse with the plasma membrane. Phafin2 could link the macropinosome membrane to the actin cytoskeleton, in line with findings that actin is required to overcome membrane tension during clathrin-mediated endocytosis^44^. Alternatively, the strong phenotype of membrane-localized Phafin2 allows for the hypothesis that Phafin2 could directly modulate the actin cytoskeleton. Actin side-binding proteins can influence the rate of actin polymerization^45^ and actin branching^46^, and high levels of Phafin2 could locally regulate actin polymerization to allow uncoating of the macropinosome. The observed phenotypes are reminiscent of the phenotypes observed after decreasing Arp2/3 activity^47,48^, raising the possibility that Phafin2 could locally inhibit actin branching. A similar mechanism has been reported for the protein CYRI-A, which transiently localizes to forming macropinosomes and locally suppresses actin dynamics by binding to Rac1^49^.

Our collective findings suggest the following model (Fig. 8f): Scission from the plasma membrane results in a nascent macropinosome that is enmeshed in actin and trapped close to the plasma membrane. In order to mature, it has to escape this meshwork by removing actin nucleating factors from its limiting membrane and shed its actin coat. Transient production of PtdIns3P and PtdIns4P by dephosphorylation of PtdIns(3,4)P_2_ and PtdIns(4,5)P_2_ triggers recruitment of Phafin2, which couples the macropinosome membrane to the actin cytoskeleton. Asymmetric recruitment and release of Phafin2 by generation and dephosphorylation of PtdIns3P or PtdIns4P generate a Phafin2 gradient which directs forces on the vesicle.

Nutrient scavenging by macropinocytosis has emerged as an important amino acid supply route in KRAS*-transformed cancers^4,37^, and we find that loss of Phafin2 impairs this process. Phafin2 is frequently amplified in cancers, raising the possibility that higher Phafin2 levels provide a growth advantage for cancer cells, possibly by enhancing macropinocytosis. It will be interesting to learn if Phafin2 cooperates with KRAS* to enhance macropinocytosis or if amplification of Phafin2 could provide an alternative, independent way for cancer cells to upregulate nutrient scavenging by macropinosome formation. Phafin2 is highly expressed in immune cells—especially dendritic cells and CD19-positive B-cells^50,51^, suggesting that Phafin2-driven macropinocytosis could help these cells with antigen sampling.

## Methods

### Cell lines

Experiments were performed in hTert-RPE1 cells (ATCC CRL-4000), MIA-PACA2 cells (ATCC CRL-1420), HT1080 cells (ATCC CCL-121), and HeLa cells (CCL-2). Cell lines were obtained from ATCC. H2B-mCherry expressing cells were generated by transfection using pH2B-mCherrry-IRES-Neo, Neomycin selection and picking of single clones. Other stable cell lines were lentivirus-generated pools. Third-generation lentivirus was generated using procedures and plasmids as previously described^52^. Briefly, tagged fusions of transgenes were generated as Gateway ENTRY plasmids using standard molecular biology techniques. From these vectors, lentiviral transfer vectors were generated by Gateway LR recombination into lentiviral destination vectors (Gateway-enabled vectors derived from pCDH-EF1a-MCS-IRES-PURO (SystemBiosciences)). VSV-G pseudotyped lentiviral particles were packaged using a third-generation packaging system (Addgene plasmids 12251, 12253, 12259). Cells were then transduced with low virus titers (multiplicity of infection < 1) and stable expressing populations were generated by antibiotic selection. Detailed cloning procedures are available from the authors.

### Stimulation of macropinocytosis in hTERT-RPE1 cells

We found that both size and initiation frequency of new macropinosomes in hTERT-RPE1 cells were strongly stimulated by addition of 50 ng/ml hepatocyte growth factor (HGF) (supplementary Fig. S5a, b, c). The effect of this stimulation persisted for up to 60 min after growth factor addition; we, therefore, performed all measurements—unless otherwise stated—in hTERT-RPE1 cells after addition of HGF. Imaging was limited to 40 min after stimulation to ensure consistent results.

### Antibodies

The following antibodies were used: Phafin2 antibody, Sigma-Aldrich (HPA024829) (WB, 1:500). FilaminA antibody, Abcam (ab76289) (WB, 1:1000). Atto-488 conjugated anti-GFP nanobody, Chromotek (gba488) (IF, 1:100). γ-Tubulin antibody, Sigma-Aldrich (T5326)(WB, 1:10000). aTubulin antibody, Sigma-Aldrich (T5168) (WB, 1:20000). Rabankyrin-5 antibody (Abnova, H0005 1479-B01P) (IF, 1:100). MBP antiserum (New England Biolabs, E3080s)(WB,1:10000). Donkey anti-mouse Alexa555 (Molecular Probes. A31570)(1:500).

### Plasmids

pEYFP-Rabankyrin-5 was a gift from Marino Zerial;^10^ EYFP was exchanged with mCherry. The following plasmids were obtained from Addgene:

Myo1E-pmAppleC1 was a gift from Christien Merrifield (Addgene #27698^30^,), pH2B-mCherrry-IRES-Neo was a gift from Daniel Gerlich (Addgene #21044^53^), pDisplay-Phuji was a gift from Robert Campbell (Addgene #61556^29^,), pEGFP-AKT-PH was a gift from Tobias Meyer (Addgene #21218^54^); EGFP was replaced with mCherry. pTagRFP-T-TAPP-2xPH was a gift from Tadaomi Takenawa^55^. pmCherry-FilaminA was a gift from Michael Davidson (Addgene plasmid # 55047), pLifeact-mTurquoise2 was a gift from Dorus Gadella (Addgene plasmid #36201);^56^ mTurquoise2 was replaced with mCherry and SNAP. pX458 and pX459 were a gift from Feng Zhang (Addgene plasmids #48138 and 62988^57^). Venus-iLID-CAAX and the SSPB-coding region were a gift from Brian Kuhlmann (Addgene #60411 and #60415)^35^. Lyn-FRB-mCherry was a gift from Robin Irvine (Addgene #38004)^58^. pET-His6-MBP was a gift of Scott Gradia (Addgene # 29708).

All other plasmids were generated using standard cloning procedures. A list of plasmids used in this study can be found in Supplementary Table 1.

### Transient transfection of cells

RPE1 cells were transfected with Fugene 6 using a ratio of 3:1 of reagent to DNA. In most cases, cells were transfected in MatTek 3.5 cm dishes using 6 ul of Fugene 6 and 2 ug of DNA. The medium was replaced 12 h after transfection to remove transfection reagent, and cells were imaged between 16 h and 20 h after transfection.

### CRISPR/Cas9-mediated deletion of Phafin2

RPE1 cells deleted for Phafin2 were generated using CRISPR/Cas9. Guide RNAs were designed using Benchling software (www.benchling.com). For deletion of Phafin2, a guide RNA binding directly at the start codon in Exon 2 and one binding after Exon 2 was chosen (gRNA1: 5'-AGGCTATTAGTGAAAGATGG-3'; gRNA2: 5'-TGGGAGTATTTAATCAGGTG-3'). As the whole ORF of Phafin2 is contained in this exon, we reasoned that the whole ORF should be excised. pX458-derived plasmids encoding both Cas9-2A-GFP and the respective gRNA were transfected using Fugene 6. 48 h post-transfection, GFP-positive cells were sorted and seeded out in several dilutions to obtain single colonies, which were picked and characterized. Clones lacking Phafin2 were identified by western blotting, and the introduced mutations were characterized by PCR, followed by cloning and sequencing. Primer sequences used for the characterization of knockout clones can be found in Supplementary Table S2. We established a Phafin2 clone lacking the whole Exon 2 in one allele and an inversion of the second allele, including a deletion of the start codon, resulting in a complete inactivation of Phafin2 (Supplementary Fig. S2). Deletion of Phafin2 in MIA-PACA2 cells followed the same protocol, but a pX459-derived plasmid and puromycin selection instead of cell sorting were used.

### siRNA-mediated depletion of Phafin2

siRNAs against Phafin2 were described before (Pedersen et al.)^11^. The following sequences were used to deplete Phafin2: siRNA 1 TGGTCAACCTTTAACTATA; siRNA2: GAAGCAAATACTAGACGTA. For siRNA transfections, 50 nM of siRNA was transfected using RNAiMax reagent. Transfected cells were analyzed 72 h after transfection; knockdown efficiency was verified by western blotting.

### Live-cell imaging

All live-cell imaging—with the exception of the light sheet microscopy—was performed on a Deltavision OMX V4 microscope (GE Healthcare) equipped with 60 × 1.42 NA objective (Olympus), three water cooled PCO.edge sCMOS cameras, a solid-state light source, and a laser-based autofocus. Environmental control was provided by a heated stage and an objective heater (20-20 Technologies). Cells were imaged in Live-Cell Imaging buffer (Invitrogen) supplemented with 20 mM glucose. To enhance the rate of macropinosome formation, cells were stimulated by adding HGF (50 ng/ml) directly before imaging. For imaging of Lifeact-SNAP, cells were stained with SiR647-SNAP (New England Biolabs) according to the manufacturer’s protocol. Images were deconvolved using softWoRx software and processed in Fiji^59,60^.

### TIRF microscopy

TIRF microscopy was performed with the Ring-TIRF mode of the OMX V4 system described above, using a 60 × 1.49 NA objective (Olympus), using 488 nm and 561 nm laser lines. All other imaging conditions were as described above.

### High-resolution light-sheet microscopy

Light-sheet microscopy was performed using a custom-built oblique plane microscope. The microscope layout followed the general plan for a stage scanning OPM microscope described in Sapoznik et al.^61^. The microscope was built around the ASI modular microscope platform. The inverted microscope consisted of an ASI MIM microscope body, an ASI FTP Z stage and a stage scanning optimized ASI X/Y stage (ASI imaging). Environmental control was provided by an Okolabs stage incubator. A 60 × 1.3 NA silicon oil immersion lens (Olympus) served as the primary objective. ASI cage elements were used to construct the rest of the optical train, consisting of a 300 mm tube lens (TL1), a 357 mm tube lens (Tl2), and a 40 × 0.95 NA air remote objective (Nikon). The image generated by the remote objective was collected by a bespoke tertiary objective (AMS-AGY v1.0, Special Optics) and focused by a 250 mm tube lens (TL3) on the sensor of an Andor Zyla 4.2 camera, resulting in a final pixel size of 91 nm. The excitation beam was coupled into the light path by a dichroic mirror placed between TL2 and the remote objective. We used a gaussian light sheet generated by an ASI light sheet generator (ASI Imaging) equipped with a cylindrical lens, coupled to a Toptica laser source (Toptica) with 405, 488, 561, and 647 nm laser lines. Image acquisition was performed by stage scanning. All hardware was synchronized by an ASI Tiger controller (ASI Imaging) and controlled by the ASI diSPIM plugin in MicroManager^62^. Deconvolution and deskewing were performed using the LLSPy software package (https://github.com/tlambert03/LLSpy/), the resulting images were visualized using Imaris.

### Structured illumination microscopy

Three dimensional SIM imaging was performed on Deltavision OMX V4 microscope with an Olympus x60 NA 1.42 objective and three PCO.edge sCMOS cameras and 488 nm and 568 nm laser lines. Cells were illuminated with a grid pattern, and for each image plane, 15 raw images (five phases and three rotations) were acquired. Super-resolution images were reconstructed from the raw image files, aligned and projected using Softworx software (Applied Precision, GE Healthcare). Images were processed in ImageJ/Fiji^60^.

### Release of mitochondrially tethered MTM1 using the RCD1 reversible dimerization system

RPE1 cells stably expressing Phafin2-GFP were transfected with a Tom20 mitochondrial targeting signal (MTS)-mTagBFP2-3xSNAPtag construct and an mCherry-FKBP-MTM1 (or MTM1 C375S) construct and incubated overnight in media containing 1 µM rCD1 (Sirius Fine Chemicals). rCD1 caused heterodimerization of the FKBP and SNAPtag moieties, anchoring MTM1 to the mitochondria. Live-cell imaging was performed to visualize Phafin2 recruitment to macropinosomes. During live-cell imaging, 1uM FK506 was added to release MTM1 into the cytoplasm, and this was monitored by mCherry fluorescence.

### Rapamycin-controlled membrane recruitment of Phafin2

Rapamycin-mediated FKBP-FRB dimerization was used for acute membrane recruitment of Phafin2^36^. Cells were transfected with Phafin2-GFP-FKBP and Lyn-FRB-mCherry. Dimerization was triggered by the addition of 250 nM of rapamycin.

### Optogenetic membrane recruitment of Phafin2

The iLID light-inducible dimerization system was used for acute, light-controlled membrane recruitment of Phafin2^35^. Cells were transfected with Phafin2-mCherry-SspB and Venus-iLID-CAAX. Dimerization was triggered by excitation of the sample with the FITC channel of the OMX widefield light source.

### Bacterial infection assays

Infection experiments were performed using *Salmonella enterica* serovar Typhimurium SL1344 expressing the pGG2 (dsRed) plasmids. The day before the infection, bacteria were grown overnight in 3 mL Lysogeny broth medium supplemented with 0.3 M NaCl at 37 °C in an orbital shaker. Growth media was supplemented with 50 mg/mL ampicillin. On the day of the infection, bacteria were subcultured at a 1:21 dilution in Lysogeny broth supplemented with 0.3 M NaCl and incubated at 37 °C for 3 h (i.e., until late exponential phase). Bacteria were then washed once and resuspended in EM buffer (120 mM NaCl, 7 mM KCl, 1.8 mM CaCl_2_, 0.8 mM MgCl_2_, 5 mM glucose, 25 mM HEPES, pH 7.3). Bacterial concentration was determined using OD600, and bacteria were diluted to the desired multiplicity of infection in warm EM buffer. For time-lapse experiments, fields of view were selected at the microscope in a 37 °C chamber. Then, the bacteria were added to the cells before the beginning of the acquisition. Time-lapse experiments were performed on an inverted Deltavision epifluorescence microscope (GE Healthcare) and a 60x oil objective. Images were deconvolved using softWoRx software

### Immunostaining and stimulation of macropinocytosis

siRNA treated cells grown on glass coverslips were stimulated with HGF (50 ng/ml) for 15 minutes in full medium (DMEM-F12, 10% FCS, Pen/Strep), fixed with 3% formaldehyde (Polysciences, 18814) for 15 min on ice, and permeabilized with 0.05% saponin (Sigma-Aldrich, S7900) in PBS. Fixed cells were then stained with a mouse anti-Rabankyrin-5 antibody (Abnova, cat no: H0005 1479-B01P) at room temperature for 1 h, washed in PBS/saponin, stained with a fluorescently labeled secondary antibody (donkey anti-mouse Alexa555, Molecular Probes CatNo.: A31570) for 1 h, washed in PBS and mounted with Mowiol (Sigma-Aldrich) containing 2 µg/ml Hoechst 33342 (Thermo Fisher Scientific, H3570). Alexa488-Phalloidin (cat no: A12379) or Alexa647-Phalloidin (cat no: A22287) were used to detect filamentous actin.

### High content imaging and analysis of Rabankyrin-5 dots and actin ruffling cells

Widefield images of siRNA transfected cells labeled for endogenous Rabankyrin-5 or filamentous actin (Alexa488-Phalloidin) were acquired automatically by an Olympus ScanR high content microscopy system using a UPLSAPO 40x objective and analyzed automatically by the ScanR software. After background subtraction, dots of Rabankyrin-5 > 30 pixels were segmented automatically by the ScanR software based on an intensity threshold, and the number of dots per cell was measured. The total number of cells was quantified automatically by the detection of Hoechst nuclear stain. Identical settings were used for all conditions within an experiment. Cells with prominent actin ruffling were scored manually.

### Confocal fluorescence microscopy

Confocal images were obtained using LSM710 confocal microscope (Carl Zeiss) equipped with an Ar-laser multiline (458/488/514 nm), a DPSS-561 10 (561 nm), a laser diode 405–30 CW (405 nm), and a HeNe laser (633 nm). The objective used was a Plan-Apochromat 63 × /1.40 oil DIC III (Carl Zeiss). Image processing was performed with basic software ZEN 2010 (Carl Zeiss) and Fiji software.

### Measurement of dextran and DQ-BSA uptake by flow cytometry

Fluid-phase uptake was assayed by measuring fluorescent dextran uptake using flow cytometry. Fluorescent dextran Alexa Fluor 488 10 kDa was added to cells (0, 5 mg/ml) stimulated with HGF and cells were incubated for 20 min at 37° C. Cells were then washed five times with prewarmed medium, trypsinized and dextran fluorescence was measured by flow cytometry. Alternatively, uptake was measured with self-quenched DQ-BSA. Cells were incubated with 10 µg/ml DQ-BSA for 30 min, washed, and chased for 1 h. Cells were then trypsinized, and DQ-BSA fluorescence was measured by flow cytometry. All flow cytometry measurements were performed using an LSR II flow cytometer equipped with 488 and 561 nm laser lines. For all flow cytometry measurements, live cells and single cells were gated, and the dextran/DQ-BSA fluorescence was measured. An exemplary gating scheme is shown in Supplementary Fig. S8.

### Dextran fluorescence by microscopy

Cells of the indicated genotypes were seeded in glass-bottomed MatTek dishes. The media was replaced by prewarmed media containing 0.5 mg/ml dextran-Alexa Fluor 488 (10 kDa) and 50 ng/ml HGF. Cells were incubated at 37°C for 30 mins. After the incubation, cells were quickly washed four times with prewarmed media, once with phosphate-buffered saline, and fixed for 10 min at room temperature using 4% paraformaldehyde in PBS. The cells were gently washed three times in PBS and the plasma membrane labeled with Wheat Germ Agglutinin-Alexa Fluor 647 (Molecular Probes) at 5 µg/ml for 10 mins in PBS. The cells were washed twice, the nuclei labeled with Hoechst 33342 (Molecular Probes), and imaged in PBS. Image z-stacks of 6 µm were acquired at an interval of 250 nm and deconvolved. One cell was measured per field of view acquired (the field of view was typically only large enough to fully fit one cell). Image stacks were z-projected using the sum of intensities. Cell outlines were segmented in an ImageJ script using the plasma membrane marker as a guide. Background values (compensation for residual nonspecific dextran and imperfect deconvolution) were obtained from an ROI in each image, excluding all cells segmented. Where the ImageJ script failed to generate a good quality segmentation, we manually generated the ROIs and took background values from a 100 × 100 pixel square outside cells. The intracellular dextran fluorescence was computed by subtracting the background from mean fluorescence within the cell and multiplying by cell area to obtain the integrated intensity of the cell. Measurement scripts are available at https://github.com/koschink/Phafin2.

### Measurement of cell proliferation under amino acid limiting conditions

MIA-PACA2 cells, either WT or Phafin2 −/− cells, were seeded in 6-well plates (50,000 cells/well). For each experiment, two technical replicates were used to minimize measurement errors. One day after seeding, the medium was changed to L-Glutamine-free DMEM supplemented with 10% dialyzed FBS and 0.5 mM Glutamine (control), 0.2 mM Glutamine (starvation), or 0.2 mM Glutamin and 2% BSA (Starvation supplemented with protein). The medium was changed every second day, and after 5 days, cells were trypsinized, resuspended in 1 ml of medium, and counted using a Coulter counter.

### Membrane scission assays

Membrane scission was assayed by measuring the fluorescence of the pH-sensitive RFP pHuji in response to pH changes. pHuji localized in sealed vesicles does not respond to changes of the extracellular pH, whereas surface-exposed fluorophores are readily quenched. Cells stably expressing Phafin2-GFP were seeded in MatTek dishes and transfected with pDisplay-pHuji. Imaging was performed in Live-Cell Imaging buffer (Invitrogen) supplemented with 20 mM glucose. After being placed on the microscope stage, a perfusion pencil (Autom8 Scientific) was placed directly on top of the cell using a micromanipulator. Short bursts of imaging buffer (140 mM NaCl, 2.5 mM KCl, 1.8 mM CaCl2, 1.0 mM MgCl2, 20 mM PIPES or HEPES) buffered to pH 5.5 and 7.5 with PIPES and HEPES, respectively, were alternatingly perfused in 5 s intervals using a gravity driven perfusion apparatus (Warner). All solutions were heated using an in-line heating element (Warner). Perfusion intervals were controlled by an Arduino microcontroller using PyFirmata and custom Python scripts (available at https://github.com/koschink/Arduino_Perfusion). Alternatively, instead of using pH changes, cells expressing the calcium sensor GCAMP6 were perfused with imaging buffers containing EGTA or Calcium using the same setup. The time point of sealing was manually determined by monitoring individual vesicles, and Phafin2 intensity was tracked as described below.

### Protein–lipid overlay assays

In vitro lipid-binding activities of Phafin2 PH and FYVE were determined by protein–lipid overlay assays^17^. Protein expression, purification, and lipid-overlay assays were performed as previously described^63^.

### Protein expression and purification

Recombinant GST- and His-MBP proteins were produced in E.coli Rosetta2(DE3) cells. Cells were grown in ZYM505 medium, and protein expression was induced by the addition of 250 µM IPTG at 20 °C overnight.

Protein purification of GST fusion proteins was performed as described^63^. Purification of His-MBP fusion proteins was performed as follows. Cells were harvested and resuspended in lysis buffer (50 mM Tris, 150 mM NaCl, 10 µM ZnCl_2_, 1 mM TCEP, 20 mM imidazole) supplemented with Complete protease inhibitors (Roche) and lysed using homogenization in an LM20 microfluidizer. Lysates were cleared by centrifugation. The cleared lysates were then loaded on HisPur columns (Pierce), and unbound proteins were removed by washing with lysis buffer. The bound protein was eluted with elution buffer (50 mM Tris, 300 mM NaCl, 10 µM ZnCl2, 1 mM TCEP, 250 mM imidazole). Protein containing fractions were pooled, dialyzed against either liposome buffer (50 m HEPES, 150 mM KCL, 100 µM ZnCl2, 1 mM TCEP) or actin assay buffer (50 mM Tris, 100 mM NaCl, 100 µM ZnCl_2_, 1 mM TCEP) overnight, snap frozen, and stored at −80 °C.

### Liposome flotation assays

Liposome flotation assays were performed similar to published procedures^21,64^. We generated liposomes with an endosome-like lipid composition and doped them with different concentrations of phosphoinositides. We chose 0.5% PtdIns3P and 5% PtdIns4P to simulate the high levels of plasma membrane-derived PtdIns4P and low levels of a phosphatase-generated PtdIns3P. PI was used as balance in liposomes doped with a single species and as negative control. Lipids (47% PC, 25% PE, 9% Cholesterol, 10% PS, 5% PI (Avanti Polar Lipids) and 0.2% NBD-PE (Thermo Fisher), all % are molar %) were dissolved in Chloroform. Then, 0.5% PtdIns3P and 5% PtdIns4P were added. In case that only one phosphoinositide was used, PI was used as balance. Liposomes containing PI only were used as negative control. The solvent was evaporated under a nitrogen stream and the lipid film dried under vacuum. The dried lipids were rehydrated in liposome buffer (50 m HEPES, 150 mM KCL, 100 µM ZnCl2, 1 mM TCEP) and multilamellar liposomes were formed by five freeze-thaw cycles. The resulting liposome mixture was then extruded by 11 passages through a 400 nm filter membrane. Recombinant MBP-Phafin2 and Phafin2 mutants were added to liposomes (1 mM lipid) in a final volume of 150 µl and incubated for 20 min at RT. To this mixture, 100 µl of 75% Sucrose in liposome buffer was added, resulting in a 30% sucrose solution. This fraction was overlaid with 200 µl 25 % sucrose in liposome buffer and 100 µl of liposome buffer without sucrose. The sample was then centrifuged at 55,000 r.p.m. (240,000 g) in a Beckman swing rotor (TLS 55) for 1 h. Successful liposome flotation was verified by visualizing NBD-PE fluorescence using a “Safelight” gel imager, and fractions (250 µl (bottom), 200 µl (middle), and 100 µl (top)) were collected from the bottom. 25 µl of the top fraction was separated using SDS-PAGE and visualized by Coomassie Brilliant Blue staining. Gels were recorded using an “Odyssey” gel imaging device (LI-COR Biotechnology). Intensities were measured using Fiji and plotted using the Seaborn Python package.

### Actin spin-down assays

The binding of Phafin2 and Phafin2 mutants to filamentous actin was measured using actin spin-down assays. His-MBP-Phafin2, Phafin2 subdomains (His-MBP-PH, His-MBP-FYVE), and isolated His-MBP were expressed as fusions in *E.coli* as described above. Directly before use, proteins were centrifuged for 1 h at 150,000 g to remove insoluble or aggregating proteins. Spin-down assays were performed with the Cytoskeleton actin spin-down kit according to the manufacturer’s instructions. Polymerized non-muscle actin was mixed with His-MBP-fusion proteins in actin polymerization buffer (final volume 100 µl). The dialysis buffer was used as balance. After incubation at RT for 1 h, filamentous actin was sedimented by ultracentrifugation (1.5 h, 150,000 g), and both pellet and supernatant fraction were analyzed by SDS-PAGE. MBP fusion was detected by western blotting with an anti-MBP antibody. Intensities were measured using Fiji and plotted using the Seaborn Python package. We quantified the intensity of the MBP fusion proteins in both the supernatant and pellet fractions ( = 100%) and plotted the fraction bound to actin in the pellet.

### Image pre-processing and data analysis

All live-cell images were deconvolved using Softworx (GE Healthcare) prior to analysis and presentation. All further image analysis and measurement steps were performed in FIJI—either by manual scoring or using custom Jython scripts. Measurement scripts are available at https://github.com/koschink/Phafin2.

### Quantification of macropinosome fusion

Macropinosome fusion was quantified by manually tracking individual macropinosomes from their formation to the endosomal phase and visually scoring fusion events.

### Tracking of macropinosomes

Newly formed macropinosomes were identified in time-lapse movies and manually tracked by using Phafin2 or membrane markers as reference. For each time point, a region of their limiting membrane was marked as the region of interest (ROI). Fluorescence intensity of a circular ROI (10 pixel diameter) surrounding the marked region was quantified in all image channels by a Jython script and measurements were exported for further analysis. All further processing steps were performed using Python scripts and the “Pandas” data analysis package. Individual tracks of macropinosomes were temporally aligned by identifying the highest Phafin2 intensity in the first 15 frames of each track (corresponding to the early phase of Phafin2 localization) and aligning all tracks to this maximum. The measurements of each track were normalized, and the mean value and the 95% confidence interval were plotted for presentation using the Seaborn Python package. Both measuring and post-processing analysis scripts are available at https://github.com/koschink/Phafin2.

### Quantification of macropinosome formation

To quantify newly formed macropinosomes (Fig. 5b, c), Phafin2 KO cells were mixed with WT cells stably expressing H2B-mCherry and transfected with GFP-2xFYVE and Lifeact-SNAP. Cells were then stimulated with HGF, and ruffling cells were imaged for 20 mins (30 s/frame). Choosing of the cells was blinded in regard to the genotype (WT cells were labeled by H2B-mCherry). Macropinosome formation was then manually quantified by scoring the number of newly formed macropinosomes—large (>1 µm diameter) 2xFYVE positive vesicles—within the imaging period.

### Quantification of successful vs. aborted macropinosome formation

Stable cell lines (WT and Phafin2 KO) expressing mNG-2xFYVE and MyrPalm-mCherry were stimulated with HGF and imaged (5 s/frame). Newly forming macropinosomes were identified as cup-shaped membrane structures using the MyrPalm-mCherry channel. Cup-shaped membrane structures successfully gaining mNG-2xFYVE and >1 µm in diameter were scored as successfully formed macropinosomes.

### Automated measurements of macropinosome size

In order to measure the size of newly formed macropinosomes, individual 2xFYVE labeled vesicles were tracked and their size was measured using Trackmate^65^ and a custom FIJI script. For each individual track, we extracted the size of the vesicle at the first time point using a custom Python script. We reasoned that extracting the first time point would provide us with the size of macropinosomes prior to fusion and fission events. We filtered out vesicles <0.64 µm² to select for macropinosomes. We then plotted the mean size of newly formed macropinosomes per cell and the size of individual macropinosomes using Seaborn. Custom measurement and analysis scripts are available at https://github.com/koschink/Phafin2.

### Statistical analysis

Statistical analysis was performed using Graphpad Prism. Student’s *t*-test was used as a measure for statistical significance when comparing two groups, ANOVA was used when comparing multiple groups. Data are always presented as mean *±* 95% CI, *p*-values < 0.05 were considered to be statistically significant. Sample sizes and *p*-values are reported in the figure legends. For visual annotation of statistical significance in graphs, the following nomenclature was used: *p < 0.05, **p < 0.01, ***p < 0.001, n.s. not statistically significant. Detailed statistical information for ANOVA is provided as ANOVA tables in the source data. In the case of some flow cytometry-based Dextran uptake assays (Supplementary Fig. S3e), measurements were normalized by setting the control measurements to one and a one-sample t-test was used to account for the lack of variability of the control. Categorical data were analyzed by Fisher’s exact test. The correlation between Filamin A removal and macropinosome survival was tested using Spearman’s correlation (Supplementary Fig. S5c).

### Software availability statement

Custom software scripts are available at https://github.com/koschink/Phafin2 (10.5281/zenodo.5512979)^66^, control software for the perfusion setup is available at https://github.com/koschink/Arduino_Perfusion.

### Reporting Summary

Further information on research design is available in the Nature Research Reporting Summary linked to this article.

## Supplementary information

Supplementary Information

Description of Additional Supplementary Files

Supplementary Movie 1

Supplementary Movie 2

Supplementary Movie 3

Supplementary Movie 4

Supplementary Movie 5

Supplementary Movie 6

Supplementary Movie 7

Supplementary Movie 8

Supplementary Movie 9

Supplementary Movie 10

Supplementary Movie 11

Supplementary Movie 12

Supplementary Movie 13

Supplementary Movie 14

Supplementary Movie 15

Supplementary Movie 16

Supplementary Movie 17

Supplementary Movie 18

Supplementary Movie 19

Supplementary Movie 20

Supplementary Movie 21

Supplementary Movie 22

Supplementary Movie 23

Supplementary Movie 24

Reporting Summary

## Data Availability

All data shown and used to generate plots, as well as detailed statistical information, accompanies this manuscript in the source data file. Uncropped western blots and gels are shown in Supplementary Fig. S9. Underlying image data are available from the corresponding authors (H.St, K.O.S) upon request. Expression data for Phafin2 is available at the public BioGPS database (https://biogps.org/gene/79666/), the cancer copy number alteration data shown in Fig. 8d and 8e are available at the cBioPortal database (https://www.cbioportal.org/). Source data are provided with this paper.

## Source data

Source Data
