# Supplementary material for: The phosphoinositide coincidence detector Phafin2 promotes macropinocytosis by coordinating actin organisation at forming macropinosomes
Source: Nat Commun. 2021 Nov 12;12:6577. doi: 10.1038/s41467-021-26775-x (PMC8590015; doi:10.1038/s41467-021-26775-x)
Supplement: Supplementary file 1 — Supplementary Information [file 41467_2021_26775_MOESM1_ESM.pdf]

Supplementary Figure S1

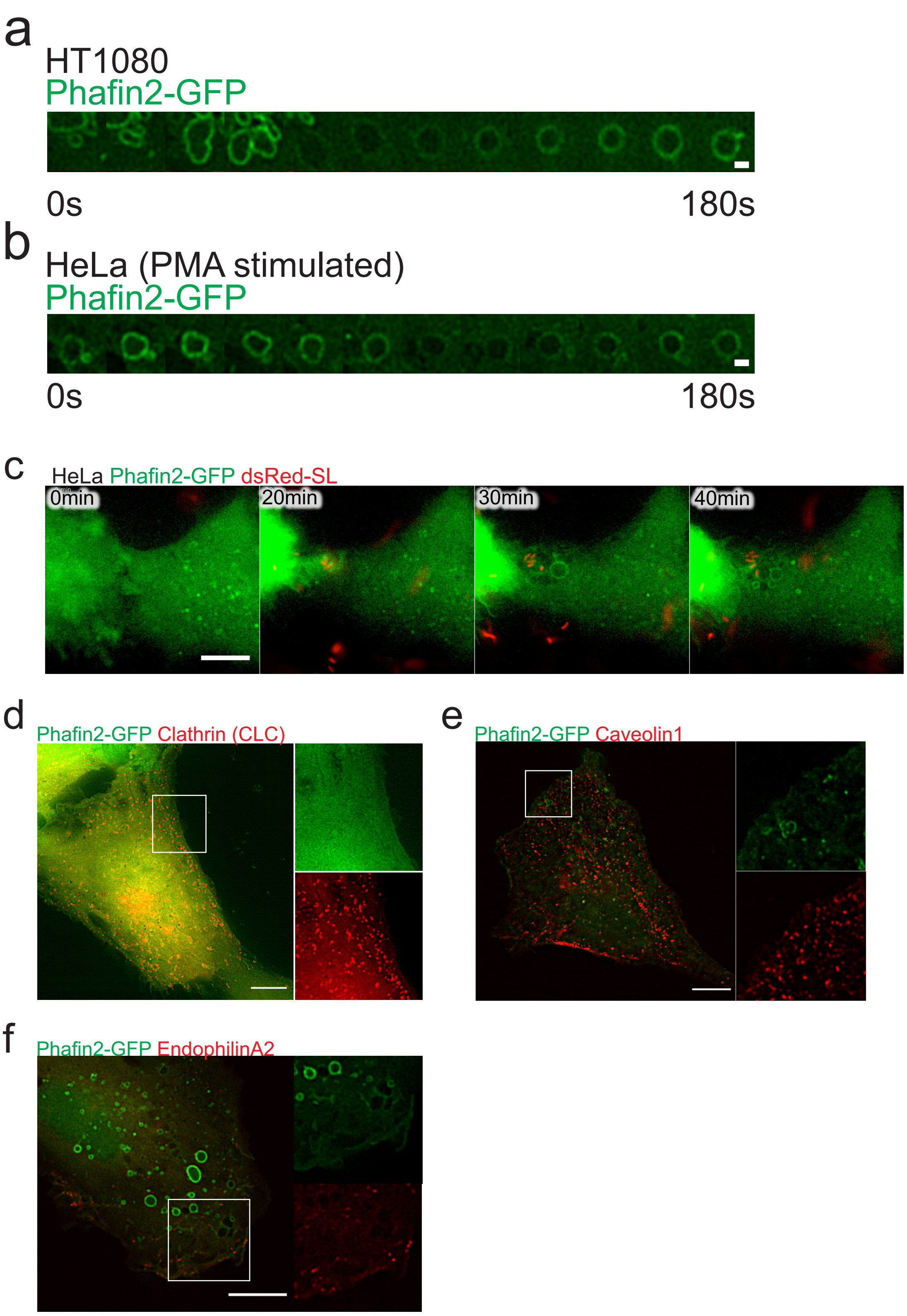

Supplementary Figure S1: a) Sequential images showing Phafin2-GFP localization to macropinosomes in HT1080 cells. Representative image from 6 cells. Scale bar: 1µm. **b)** Sequential images showing Phafin2 localization to macropinosomes in HeLa cells. Representative image from 10 cells. Scale bar: 1µm. **c)** Phafin2 localizes to *Salmonella*-induced macropinosomes and *Salmonella*-containing vacuoles in HeLa cells. Scale bar: 10 µm. Representative image for 9 cells. **d)** TIRF image showing Phafin2 localization in relation to mCherry-tagged clathrin (CLC). Phafin2 does not localize to clathrin-coated pits. Representative image from 6 cells. Scale bar: 10µm. e) Phafin2 localization in relation to mRFP-tagged Caveolin1. Scale bar: 10 µm. Representative image from 5 cells f) Phafin2 localization in relation to mCherry-tagged Endophilin A2. Representative image from 5 cells. Scale bar: 10 µm.

Supplementary Figure S2

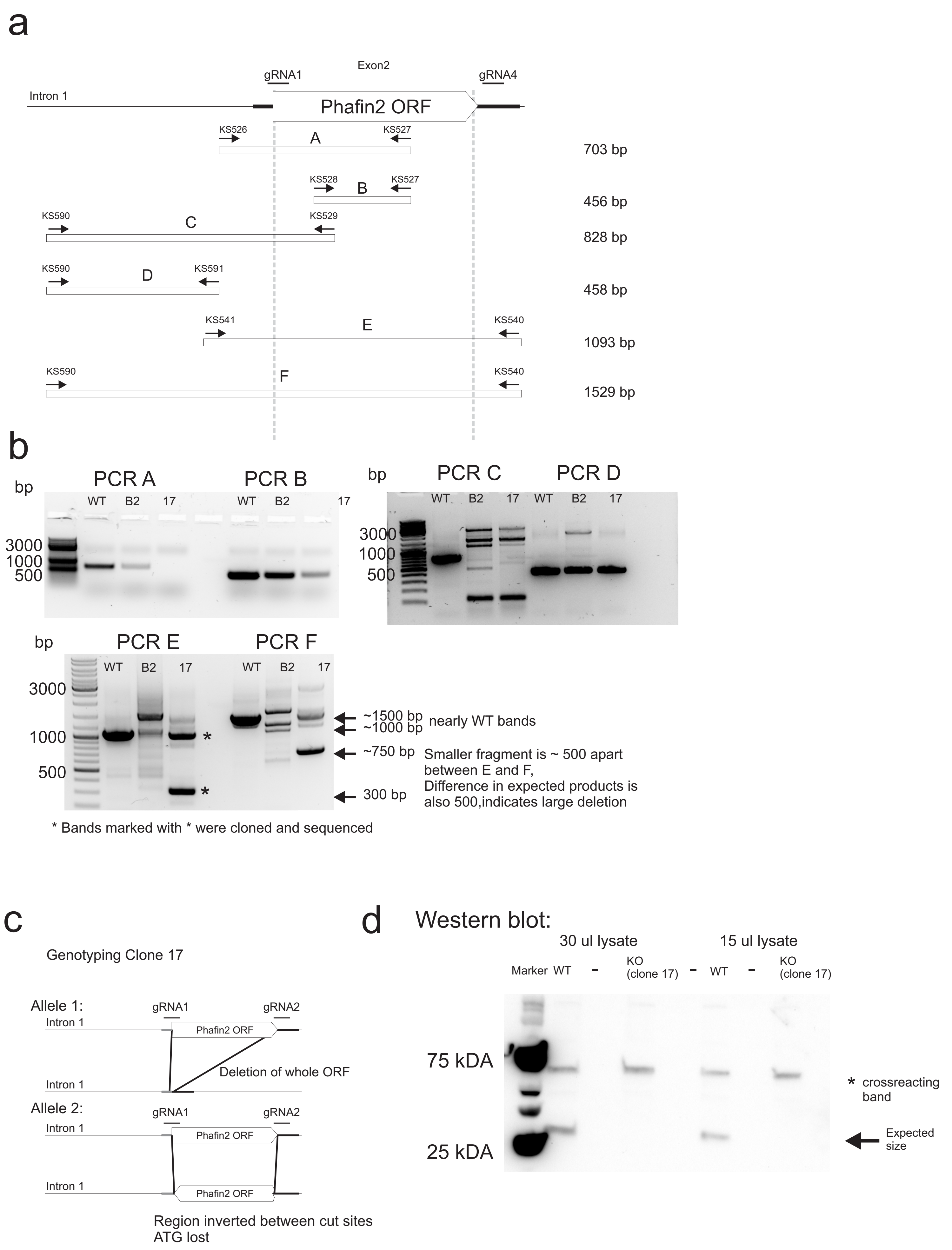

Supplementary Figure S2: Characterization of CRISPR-generated Phafin2 KO cell lines (related to Figure 5). **a)** Schematic display of the Intron-Exon structure of Phafin2, localization of gRNAs, primer combinations and expected product sizes used to characterize Phafin2 KO cell lines. **b)** PCR products from Phafin2 KO cell lines. Representative image of 3 experiments. **c)** Genomic organization of the used Phafin2 -/- cell line. d) Western blot showing complete absence of Phafin2 in the used Phafin2 -/- cell line. Representative image of 3 experiments.

Supplementary Figure S3

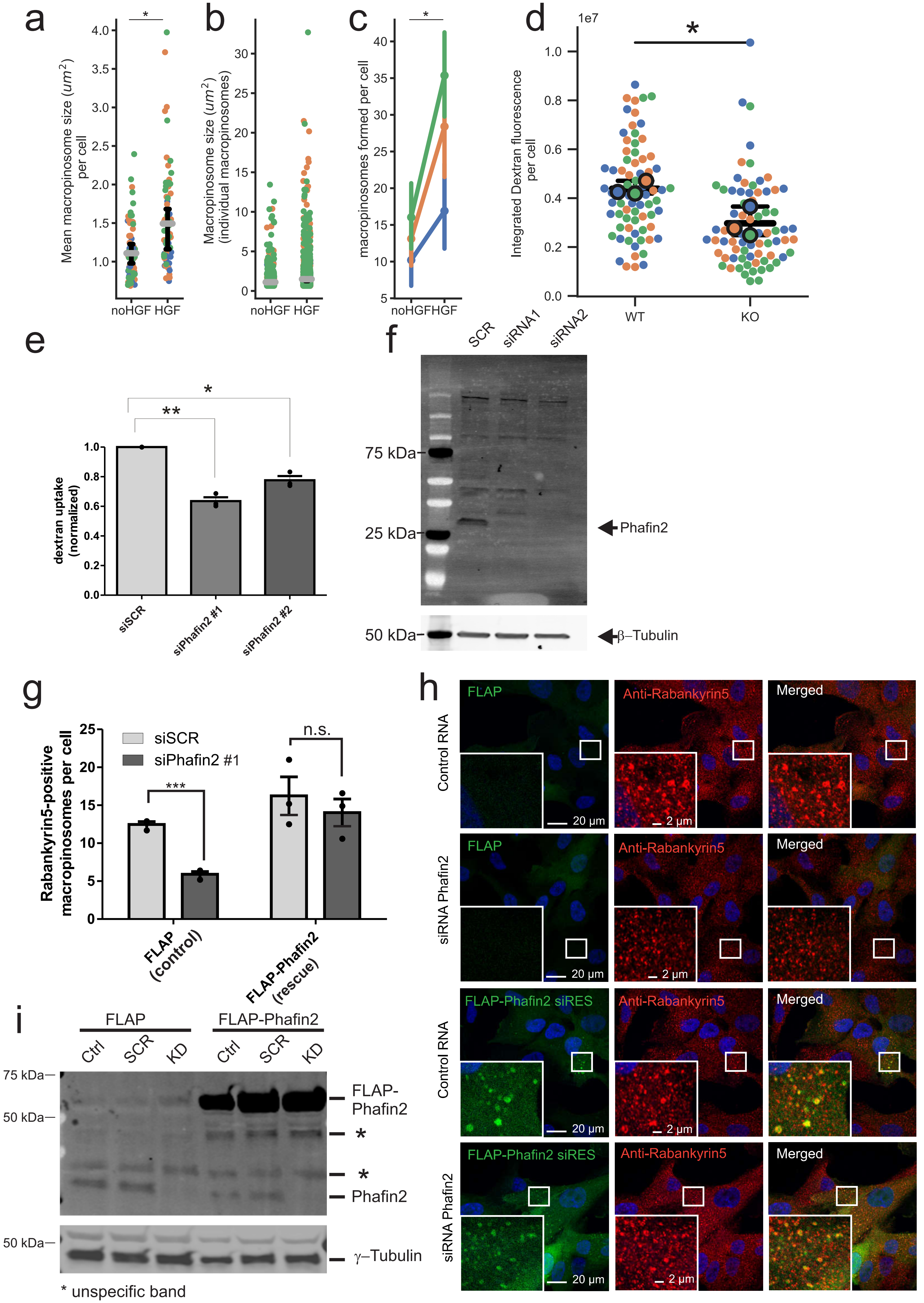

Supplementary Figure S3: Depletion of Phafin2 impairs macropinocytosis and macropinosome maturation (related to Figure 5). **a)** Stimulation of RPE1 cells with HGF increased the mean macropinosome size. n=3 experiments, with 21, 28 and 25 cells per experiment. Mean + 95% CI. Paired two-sided t-test, p=0.0425. **b)** Stimulation of RPE1 cells with HGF leads to the formation of large macropinosomes. Shown is the distribution of individual macropinosome sizes from the experiments shown in S3a. Mean + 95% CI. **c)** Stimulation of RPE1 cells with HGF increases the number of newly-formed macropinosomes per cell. n=3 experiments. Mean + 95% CI. Paired two-sided t-test, p=0.0175. **d)** Microscopic analysis of dextran uptake in wild-type and Phafin2 KO cells. n=3 experiments, with 25, 24 and 25 cells per experiment and condition. mean+95%CI, two-sided t-test, p=0.022. **e)** Depletion of Phafin2 by two individual siRNAs leads to reduced dextran uptake in HGF-stimulated cells. n=3 experiments, mean+SEM, two-sided one-sample t-test with a theoretical mean of 1, p=0.0051 (siRNA1), p=0.0161 (siRNA2). **f)** Western blot showing depletion of Phafin2 by siRNA treatment. Representative blot for n=3 experiments. **g)** High content imaging shows that depletion of Phafin2 by siRNA results in fewer large Rabankyrin5-positive macropinosomes (>30 pixel) per cell. This can be rescued by expression of a siRNA-resistant Phafin2 construct. n=3 experiments, in total 2597, 3194, 3470 and 3357 cells for siSCR(control), siPhafin2(control), siSCR(rescue) and siPhafin2(rescue). Shown are mean+SEM, two-sided t-test, p=0,0002. **h)** Depletion of Phafin2 results in fewer Rabankyrin5-labelled large macropinosomes per cell. Representative confocal images from experiment S3b. Scale bar: 20 µm, inset: 2µm. **i)** Western blot showing depletion of Phafin2 and re-expression of siRNA-resistant Phafin2 (corresponds to S3c,d). Representative blot for n=3 experiments. Statistics source data for Fig S3a, S3c, S3d, S3e, and S3g can be found in the Source Data table.

Supplementary Figure S4

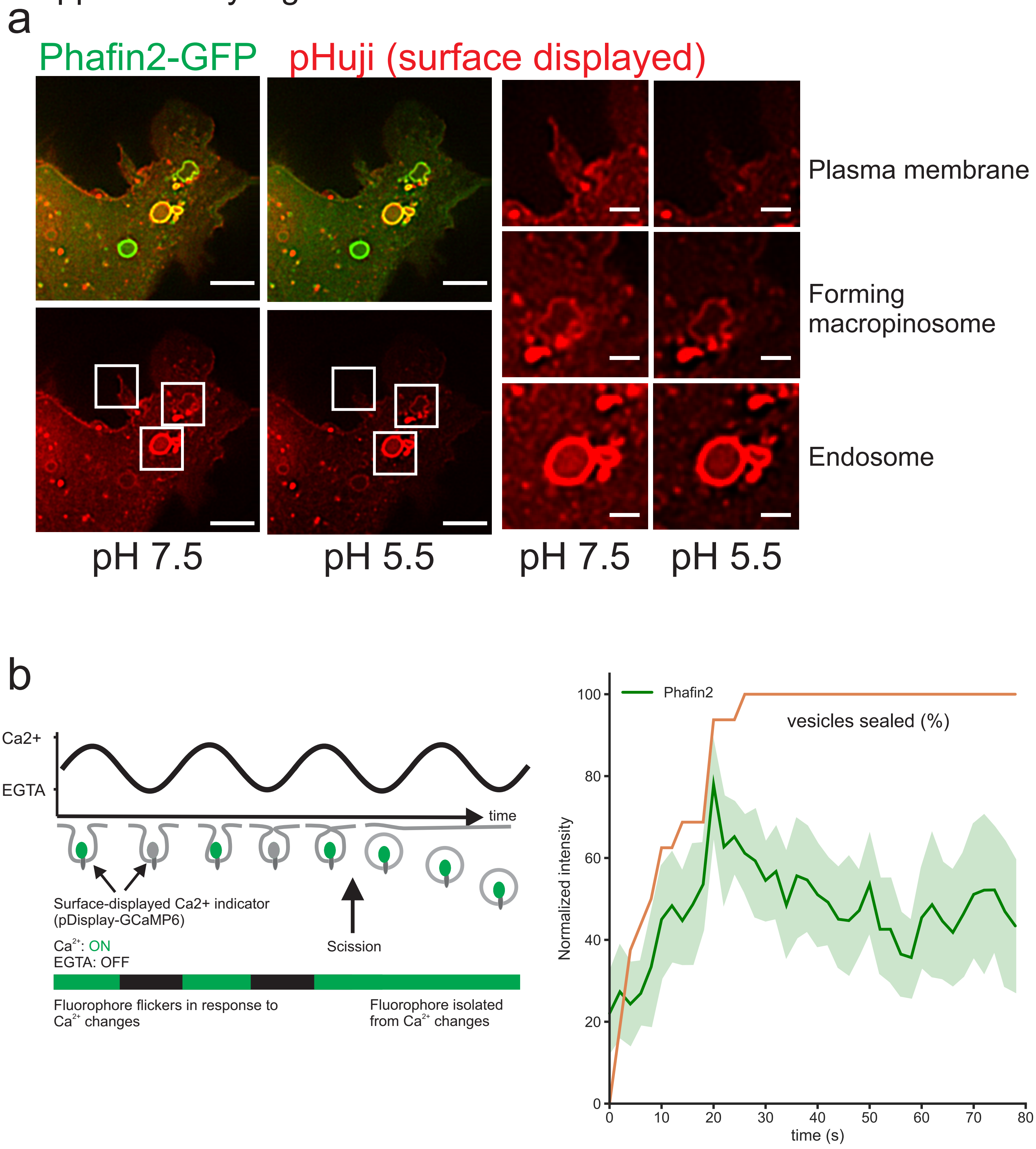

Supplementary Figure S4: Phafin2 arrives after scission from the plasma membrane (related to Figure 5). **a)** Control experiments for pH-based membrane scission assays. Plasma membrane displayed pHuji gets effectively quenched by lowering the pH of the surrounding medium. In contrast, pHuji in both newly-formed Phafin2-labelled macropinosomes and endosomal stages is not quenched. Representative image from 10 images. Scale bar: 5 µm, inset 1 µm. **b)** Calcium-based membrane scission assay. Surface-expressed calcium sensitive fluorescent protein GCAMP can be quenched and unquenched by EGTA and Ca2-containing buffers. Phafin2 arrival occurs after sealing of the vesicle. n=16 macropinosomes, mean+95% CI. Statistics source data for Fig S4b can be found in the Source Data table.

Supplementary Figure S5

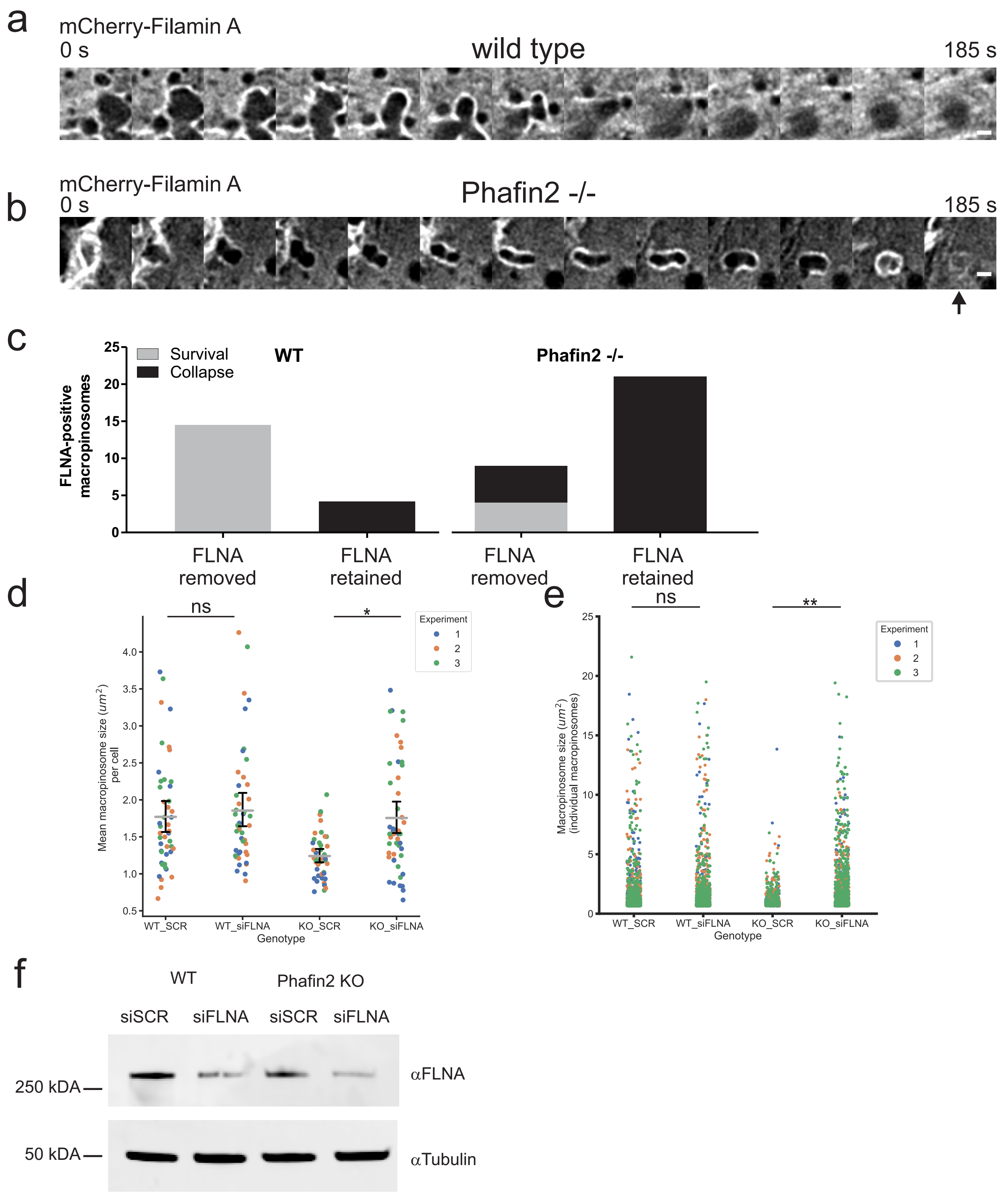

Supplementary Figure S5: a) Image series showing that newly formed macropinosomes are initially coated in FilaminA, which is removed during their transition to later macropinosomes in wild-type cells. Representative image for 18 macropinosomes. Scale bar: 1µm. **b)** Image series showing that in Phafin2 knockout cells, newly formed macropinosomes collapse before the FilaminA coat is removed. Representative image for 30 macropinosomes. Scale bar: 1µm. **c)** Analysis of macropinosome dynamics and survival in wild-type and Phafin2 -/- cells. Forming macropinosomes were scored for shedding of FilaminA and their transition to round macropinosomes or back-fusion to the plasma membrane. Data from 18 (WT) and 30 (KO) macropinosomes. **d)** Knockdown of FilaminA restores macropinosome size in Phafin2 knockout cells. n=3 experiments, mean + 95% CI, ANOVA with Tukeys post-test, p=0.0121. **e)** Phafin2 KO cells depleted for FilaminA can form large macropinosomes. Plotted is the size of individual macropinosomes (n=3, mean + 95% CI, ANOVA with Tukeys post-test, p=0.0098. **f)** Western blot showing the efficiency of the siRNA treatment in (d) and (e). Statistics source data for Fig S5c, S5d, S5e can be found in the Source Data table.

Supplementary Figure S6

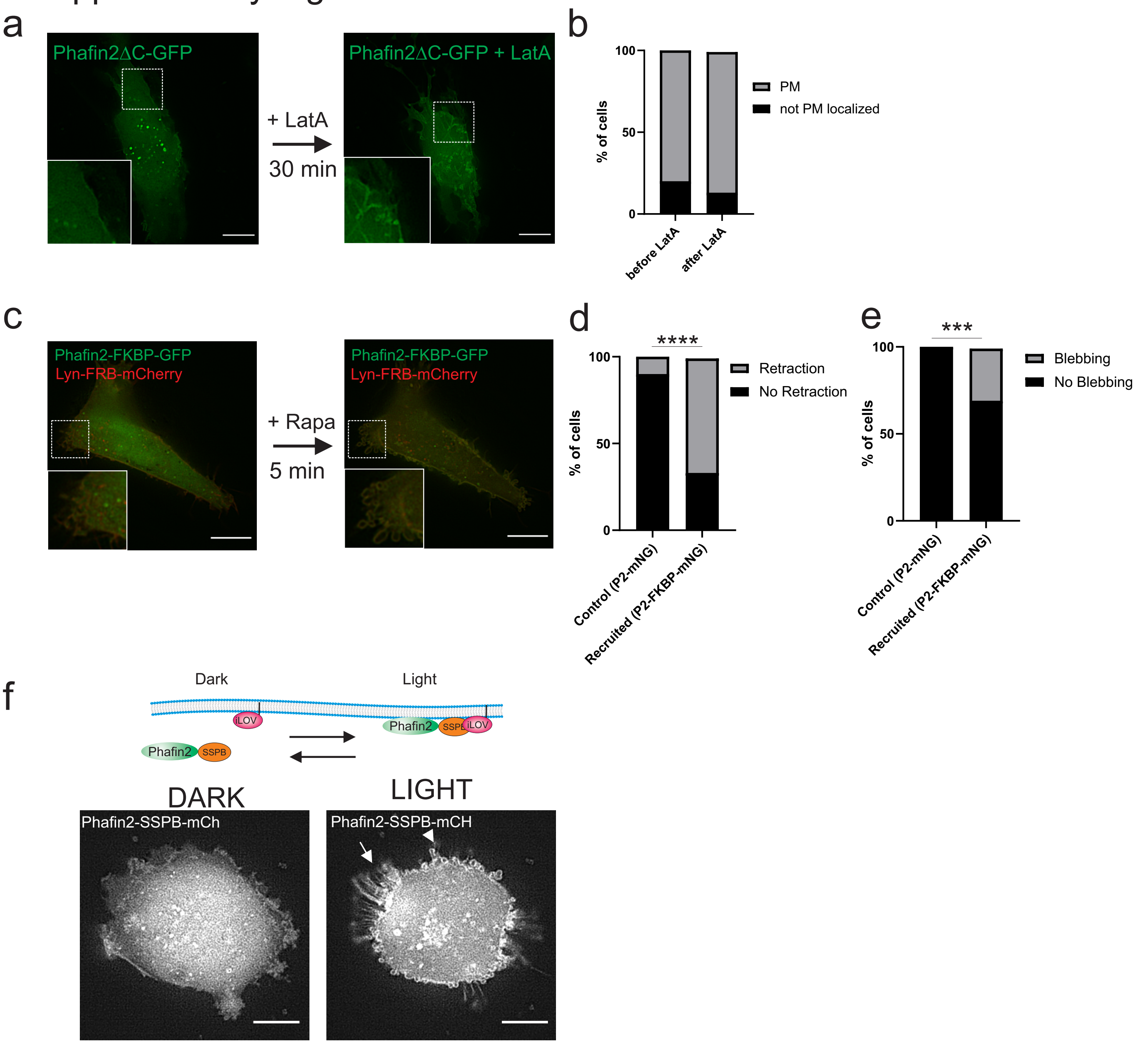

Supplementary Figure S6: Plasma membrane recruitment of Phafin2 triggers cell retractions and membrane blebbing. **a)** Membrane recruitment of Phafin2ΔC does not require actin. Representative images of cells before and after treatment with 250nM LatrunculinA. Scale bar: 10 µm. **b)** Quantification of the membrane association of Phafin2ΔC before and after LatrunculinA treatment. n=15 cells, two-sided Fisher's exact test, p=0.9999 (not significant). **c)** Rapamycin-mediated plasma membrane recruitment of Phafin2 triggers membrane blebbing. Representative images for 33 cells. Scale bar: 10 µm. **d)** Quantification of cell retraction after rapamycin-mediated membrane recruitment of Phafin2. n= 30 cells (control), 33 cells (treated). Two-sided Fisher's exact test, p<0.0001. **e)** Quantification of membrane blebbing after rapamycin-mediated membrane recruitment of Phafin2. n= 30 cells (control), 33 cells (treated). Two-sided Fisher's exact test, p=0.001. **f)** Time-lapse images of cells expressing light-dimerizable Phafin2 (Phafin2-SSBP and iLID-CAAX) and Lifeact before and after light-induced plasma membrane recruitment. Arrowheads indicate membrane blebs, arrows cell retraction. Representative image for n=25 cells. Scale bar: 10 µm. Statistics source data for Fig S6b, S6d, S6e can be found in the Source Data table.

Supplementary Figure S7

a

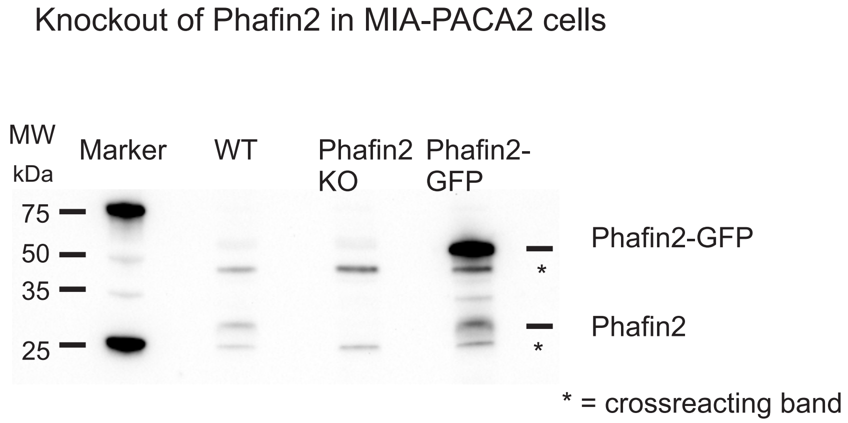

Supplementary Figure S7: a) Knockout of Phafin2 in MIA-PACA2 cells. a) Western blot against Phafin2. Shown are wild-type, Phafin2 -/- and Phafin2-GFP expressing cells. Representative image for 3 experiments.

Supplementary Figure S8

Gating strategy used to measure dextran uptake

(Figure 5a, Supplementary Figure 3a)

BD FACSDiva 8.0.3

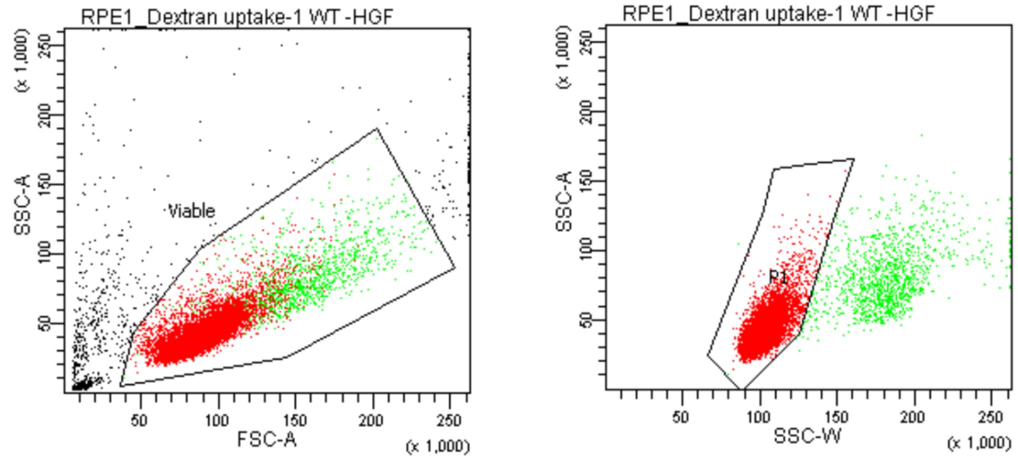

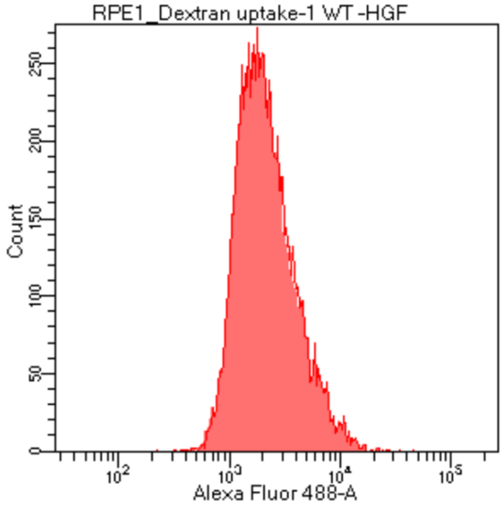

Tube: 1 WT -HGF

Population	#Events	%Parent	%To
All Events	12,333	####	10
Viable	11,329	91.9	9
P1	10,000	88.3	8

Experiment Name:	2019-10-01_Dextran
Specimen Name:	RPE1_Dextran uptake
Tube Name:	1 WT -HGF
Record Date:	Oct 3, 2019 2:49:10 PM

Population	#Events	Alexa Fluor 488-A Mean	Alexa Fluor 488-A Median	Alexa Fluor 488-A SD
All Events	12,333	3,269	1,938	10,970
Viable	11,329	3,301	2,016	10,684
P1	10,000	3,074	1,885	11,032

Median fluorescence was used for statistical analysis

Supplementary Figure 8: a) Exemplary gating strategy for flow cytometry data. Viable cells were gated in by FSCA/SSC-A scatter plots; from this gate, single cells were gated by SSC-W/ SSC-A scatter plots. No further gating was performed, all cells in the single cell gate were measured for dextran fluorescence

Supplementary Figure 9
Uncropped gels and Western Blots

Figure 4i

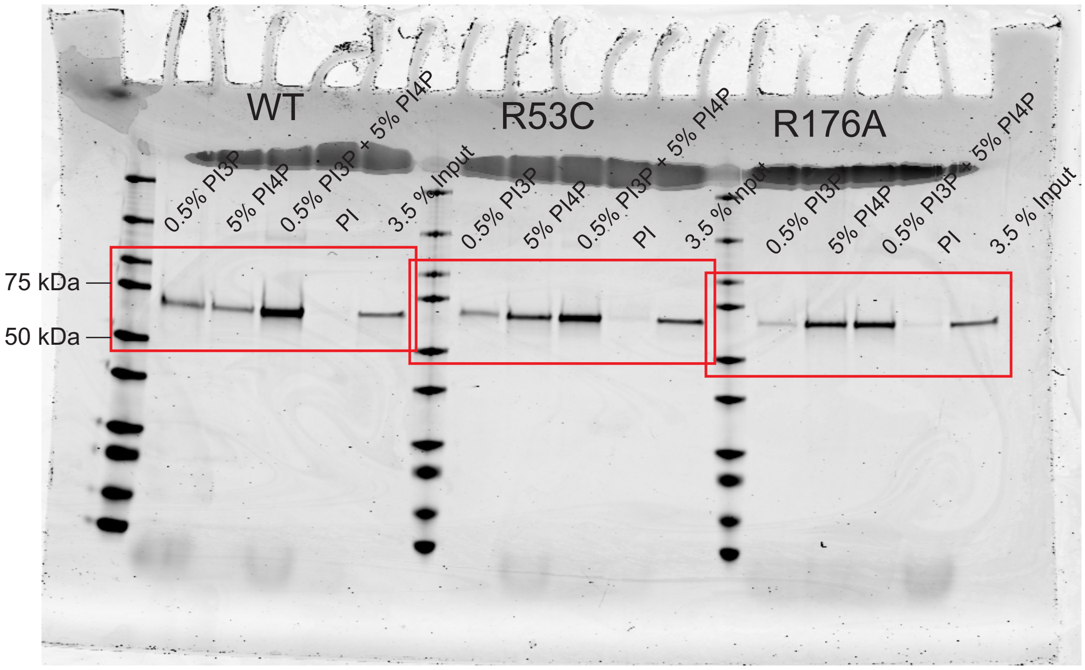

Figure 7c

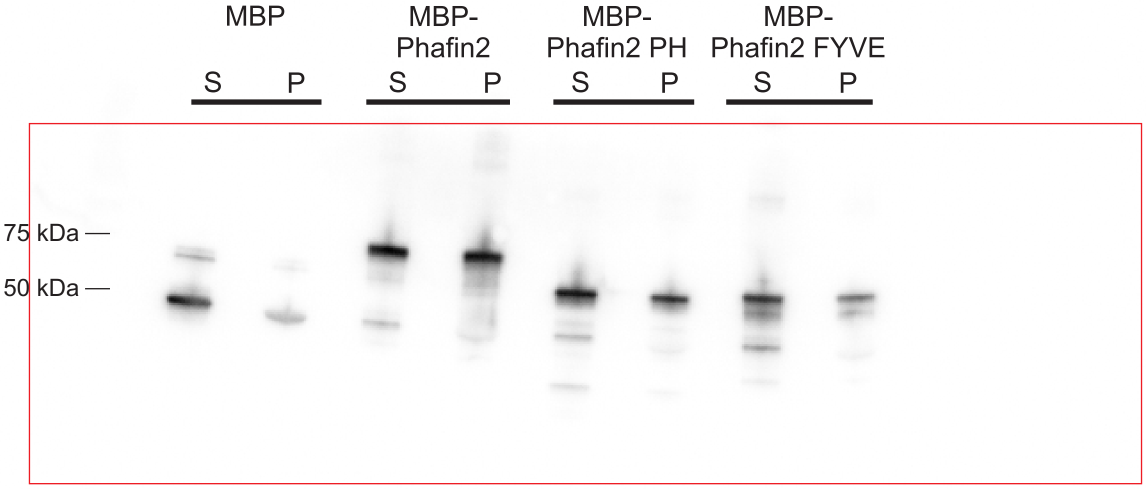

Supplementary Figure 9: Figure showing uncropped images of all gels and Western blots. Red rectangles indicate the cropped areas shown in the manuscript.

Supplementary Figure 9 (continued)
Uncropped gels and Western Blots

Supplementary Figure 1b

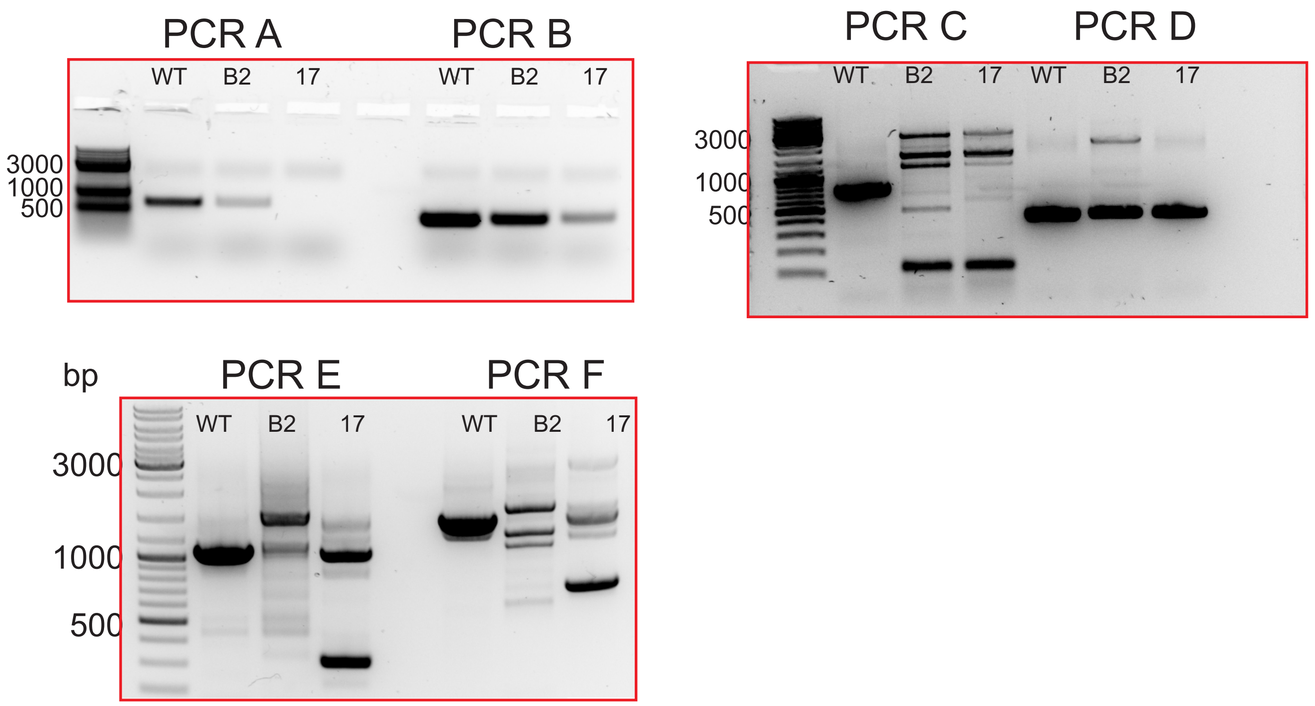

Supplementary Figure 1D

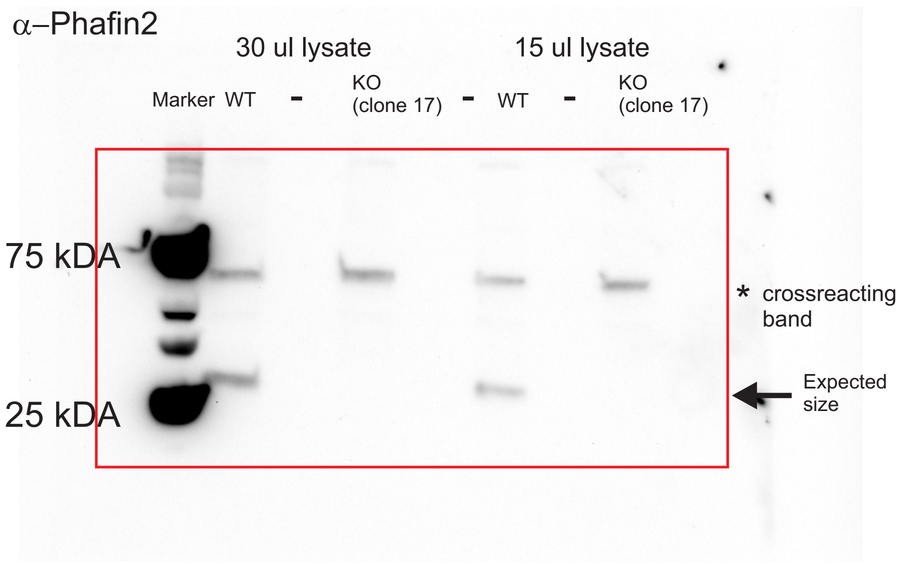

Supplementary Figure 9: Figure showing uncropped images of all gels and Western blots. Red rectangles indicate the cropped areas shown in the manuscript.

Supplementary Figure 9 (continued)
Uncropped gels and Western Blots

Supplementary Figure 3f

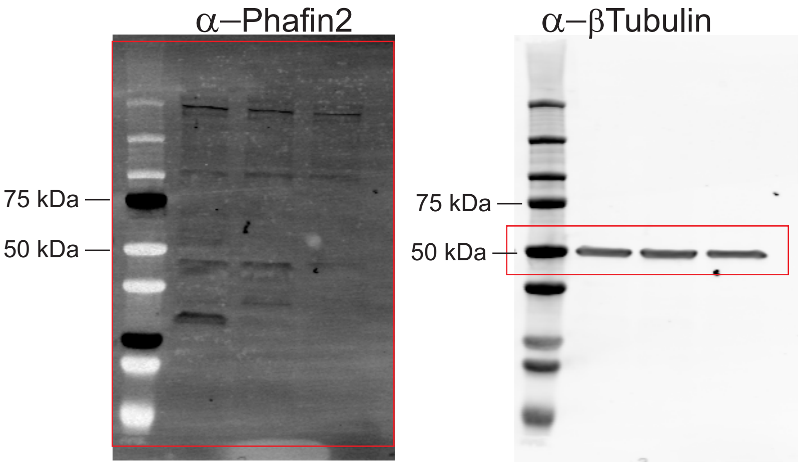

Supplementary Figure 3i

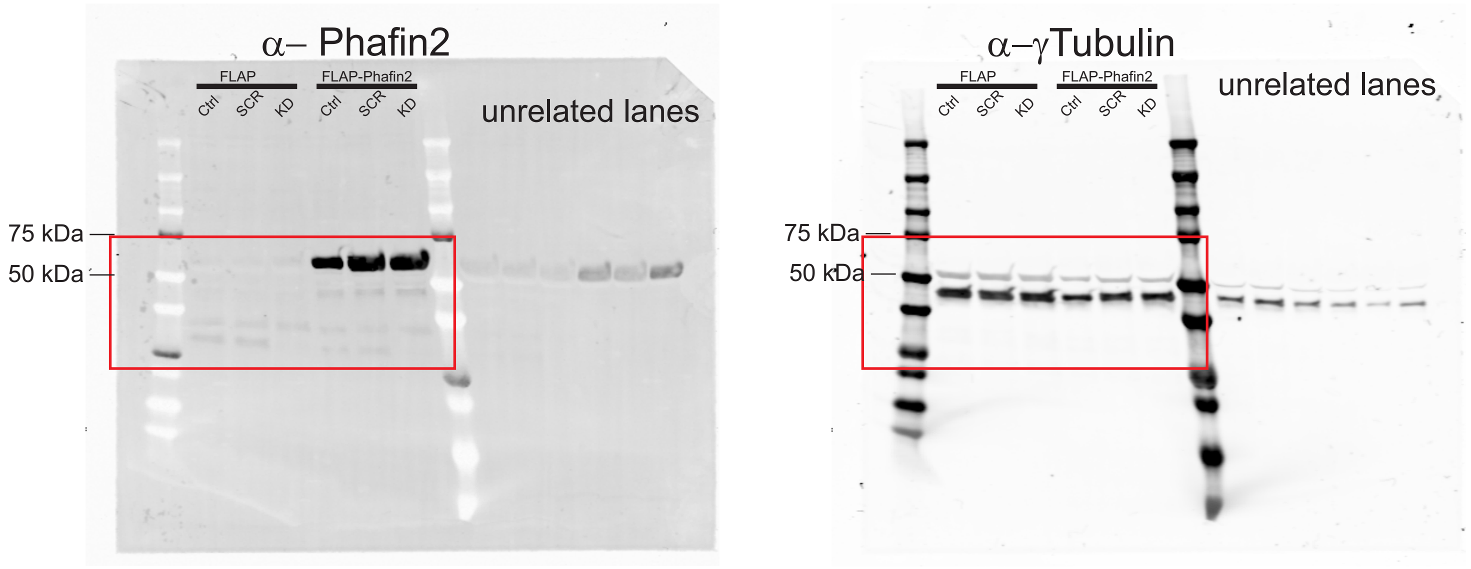

Supplementary Figure 9: Figure showing uncropped images of all gels and Western blots. Red rectangles indicate the cropped areas shown in the manuscript.

Supplementary Figure S9 (continued)
Uncropped gels and Western Blots

Supplementary Figure S5f

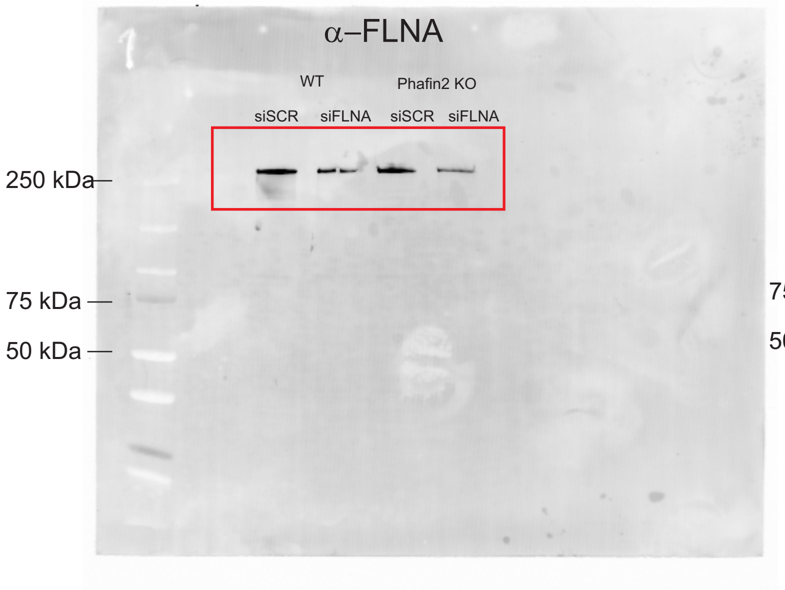

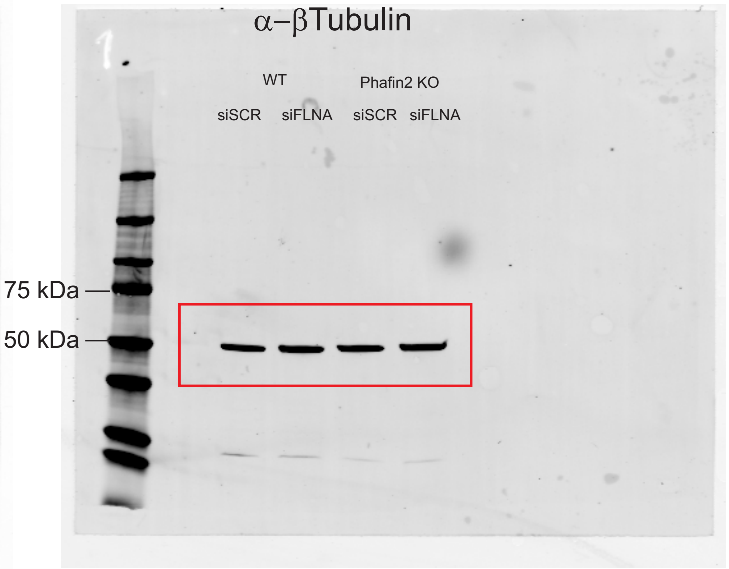

Supplementary Figure S7a

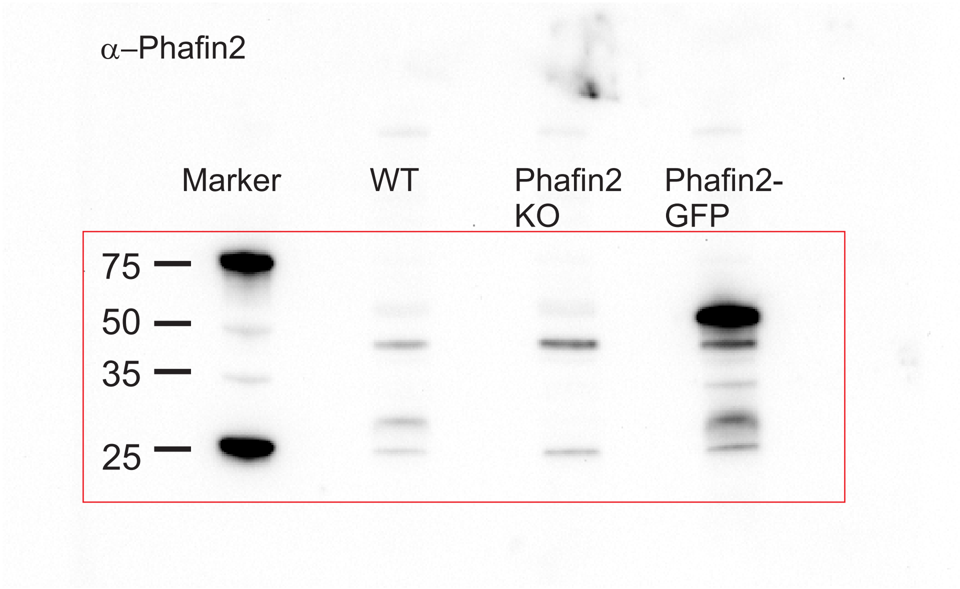

Supplementary Figure 9: Figure showing uncropped images of all gels and Western blots. Red rectangles indicate the cropped areas shown in the manuscript.

Table S1: Plasmids used in this study

Plasmids	Origin	Additional information
pCDH-EF1a-Phafin2-GFP_IRES_Puro	This study	n/a
pmCherry-N1-Phafin2(R53C)	This study	n/a
pmCherry-N1-Phafin2(R176A)	This study	n/a
pCDH-EF1a-FLAP-Phafin2(siRNA-	This study	n/a
pCDH-EF1a-FLAP _IRES_BLAST	This study	n/a
pEGFP -Phafin2	This study	n/a
pPhafin2 ΔN-GFP	This study	n/a
pEGFP -Phafin2 ΔPH	This study	n/a
pEGFP -Phafin2 ΔFYVE	This study	n/a
pPhafin2DC-GFP	This study	n/a
pMyrPalm-mCherry	This study	n/a
pDisplay-Phuji	Shen et al.. 2014 [1]	Addgene plasmid 61556
pmCherry-APPL1	This study	
pmCherry-Rabankyrin5	Marino Zerial: Schnatwinkel et al. 2004 [2]	EYFP-Rabankyrin-5, exchanged with mCherry
pmCherry-Rab5	This study	
pmCherry-Rab31	This study	
pmApple-Myo1E	Taylor et al.. 2011 [3]	Addgene plasmid # 27698
pmCherry-N1-SidC	This study	
pmCherry-AKT PH	Raucher et al.. 2000 [4]	Addgene plasmid #21218, GFP exchanged with mCherry
pTagRFP-TAPP-2xPH	Oikawa et al. 2008 [5]	n/a
pmCherry-2xFYVE	Sneeggen et al. 2019 [6]	n/a
pEGFP-2xFYVE	Gillooly et al.. 2001 [7]	n/a
pLifeact-SNAP	Goedhart et al.. 2012 [8]	Addgene plasmid 36201, Exchanged mTurquoise2 with SNAP
pLifeact-mCherry	Goedhart et al.. 2012 [8]	Addgene plasmid 36201, Exchanged mTurquoise2 with mCherry
pmCherry-FilaminA	Michael Davidson, unpublished	Addgene plasmid # 55047
pX458	Ran et al., 2013 [9]	Addgene plasmid # 48138
pX458-Phafin2-gRNA1	This study	n/a
pX458-Phafin2-gRNA2	This study	n/a
pGEX6P-1-Phafin2 PH	This study	n/a
pGEX6P-1-Phafin2 FYVE	This study	n/a
pmTurquoise2-FilaminA	This study	n/a
pPhafin2-mNeonGreen	This study	n/a
pET-His6-MBP	Scott Gradia, unpublished	Addgene plasmid # 29708
pET-His6-MBP- Phafin2	This study	n/a
pET-His6-MBP- Phafin2 (R53C)	This study	n/a
pET-His6-MBP- Phafin2 (R176A)	This study	n/a
pET-His6-MBP- Phafin2 PH	This study	n/a
pET-His6-MBP- Phafin2 FYVE	This study	n/a
pmCherry-C1-SNX5	This study	n/a
pmNeonGreen-2xFYVE	This study	n/a
pMitoSNAP	This study	n/a
pmCherry-FKBP-MTM1	This study	n/a
pmCherry-FKBP-MTM1 (C375S)	This study	n/a
pPhafin2(1X-PH)-EGFP	This study	n/a
pmCherry-C1-Phafin2(2xPH)	This study	n/a
pLL7.0: Venus-iLID-CAAX (from	Guntas et al. 2015 [10]	Addgene plasmid # 60411
pPhafin2-mCherry-SSPB	This study	n/a
Phafin2-GFP-FKBP	This study	n/a
pLyn-FRB-mCherry	Hammond et al. 2012 [11]	Addgene plasmid # 38004
pSidM-FYVE	This study	n/a
pSidM-FYVE(R176A)	This study	n/a

Table S2: Primer sequences used in this study

Number	Name	Sequence	Use	Figure
KS526	Phafin2_Test-PCR_for	GCCTTTGTGTAAAAGCAATGATTTGTTAGC	Verification of Phafin2 KO	S2a,b
KS527	Phafin2_Test-PCR_rev	TGGCTGGGAAGAAGAAATCTCTTTTCAGAGC	Verification of Phafin2 KO	S2a,b
KS528	Phafin2_Test-PCR_int_for	GTTGTGCAGGAAAAAGCCCAAAGCAAGGC	Verification of Phafin2 KO	S2a,b
KS529	Phafin2_Test-PCR_int_rev	GCCTTGCTTTGGGCTTTTTCCTGCACAAC	Verification of Phafin2 KO	S2a,b
KS540	Phafin2_Test-PCR_for #2	CAGGCAAATTCATGGCTAGAA	Verification of Phafin2 KO	S2a,b
KS541	Phafin2_Test-PCR_rev #2	TATGGTGCTACGTGGAGATG	Verification of Phafin2 KO	S2a,b
KS590	Phafin2_Test-PCR_for #3	TGAAGACTGACTTATGGTGAGG	Verification of Phafin2 KO	S2a,b
KS591	Phafin2_ver2_rev_5'_internal	TTTATCATCTCCACGTAGCACC	Verification of Phafin2 KO	S2a,b

Supplementary references

1. Shen, Y., Rosendale, M., Campbell, R.E. & Perrais, D. pHuji, a pH-sensitive red fluorescent protein for imaging of exo- and endocytosis. J Cell Biol 207, 419-432 (2014).
2. Schnatwinkel, C. *et al.* The Rab5 effector Rabankyrin-5 regulates and coordinates different endocytic mechanisms. *PLoS biology* **2**, E261 (2004).
3. Taylor, M.J., Perrais, D. & Merrifield, C.J. A high precision survey of the molecular dynamics of mammalian clathrin-mediated endocytosis. *PLoS biology* **9**, e1000604 (2011).
4. Raucher, D. *et al.* Phosphatidylinositol 4,5-bisphosphate functions as a second messenger that regulates cytoskeleton-plasma membrane adhesion. *Cell* **100**, 221-228 (2000).
5. Oikawa, T., Itoh, T. & Takenawa, T. Sequential signals toward podosome formation in NIH-src cells. *The Journal of cell biology* **182**, 157-169 (2008).
6. Sneeggen, M. *et al.* WDFY2 restrains matrix metalloproteinase secretion and cell invasion by controlling VAMP3-dependent recycling. *Nature Communications (in press)* (2019).
7. Gillooly, D.J. *et al.* Localization of phosphatidylinositol 3-phosphate in yeast and mammalian cells. *EMBO J* **19**, 4577-4588 (2000).
8. Goedhart, J. *et al.* Structure-guided evolution of cyan fluorescent proteins towards a quantum yield of 93%. *Nat Commun* **3**, 751 (2012).
9. Ran, F.A. *et al.* Genome engineering using the CRISPR-Cas9 system. *Nat Protoc* **8**, 2281-2308 (2013).
10. Guntas, G. *et al.* Engineering an improved light-induced dimer (iLID) for controlling the localization and activity of signaling proteins. *Proc Natl Acad Sci U S A* **112**, 112-117 (2015).
11. Hammond, G.R. *et al.* PI4P and PI(4,5)P2 are essential but independent lipid determinants of membrane identity. *Science* **337**, 727-730 (2012).
