# Supplementary material for: The phosphoinositide coincidence detector Phafin2 promotes macropinocytosis by coordinating actin organisation at forming macropinosomes
Source: Nat Commun. 2021 Nov 12;12:6577. doi: 10.1038/s41467-021-26775-x (PMC8590015; doi:10.1038/s41467-021-26775-x)
Supplement: Supplementary file 2 — Description of Additional Supplementary Files [file 41467_2021_26775_MOESM2_ESM.pdf]

Supplementary movie legends

File Name: Supplementary Movie 1
Description: Phafin2-GFP localizes to macropinosomes directly after cup closure, indicated by the plasma membrane probe MyrPalm-mCherry (related to Figure 1). Images were acquired every 3s.

File Name: Supplementary Movie 2
Description: The first localization of Phafin2 occurs prior to recruitment of the early endosomal protein APPL1 (related to Figure 2). Images were acquired every 5s.

File Name: Supplementary Movie 3
Description: A functional PH domain is required for localization to newly formed macropinosomes (related to Figure 3). While wild-type Phafin2 shows a biphasic localization, a mutant with an inactive PH domain does not show the first localization phase on newly formed macropinosomes, but is still able to localize to the second, endosomal phase. Images were acquired every 5s.

File Name: Supplementary Movie 4
Description: A functional Phafin2 FYVE domain is required for membrane association (related to Figure 3). Wild-type Phafin2 shows a biphasic localization, whereas a FYVE domain mutant shows a completely cytosolic localization. Images were acquired every 5s.

File Name: Supplementary Movie 5
Description: The first phase of Phafin2 localization is independent of Vps34 activity (related to Figure 4). Inhibition of Vps34 by the VPS34 inhibitor SAR405 does not affect the first transient localization phase of Phafin2 to nascent macropinosomes, but completely abolishes the second localization phase to endosomal stages. Images were acquired every 5s.

File Name: Supplementary Movie 6
Description: Release of mitochondrially tethered MTM1 results in loss of Phafin2 localization from forming macropinosomes (related to Figure 4). Images were acquired every 5s.

File Name: Supplementary Movie 7
Description: Release of mitochondrially tethered catalytically inactive MTM1(C375S) does not affect Phafin2 localization (related to Figure 4). Images were acquired every 5s.

File Name: Supplementary Movie 8
Description: A double PH domain of Phafin2 localizes to the plasma membrane and forming macropinosomes, but not to endosomes (related to Figure 4). Images were acquired every 5s.

File Name: Supplementary Movie 9
Description: Macropinosome formation in wild-type and Phafin2 KO cells (related to Figure 5). Wild-type cells show robust formation of new macropinosomes, as indicated by formation of large,

2xFYVE-positive vesicles; Phafin2 KO cells only rarely form large macropinosomes, but rather show formation of multiple small vesicles. Images were acquired every 30s.

File Name: Supplementary Movie 10
Description: In wild-type cells, macropinosomes establish endosomal identity and gain the endosomal lipid PtdIns3P (related to Figure 5). Images were acquired every 5s.

File Name: Supplementary Movie 11
Description: Macropinosomes in Phafin2 knockout cells frequently collapse before they establish an endosomal identity (related to Figure 5). Images were acquired every 5s.

File Name: Supplementary Movie 12
Description: Macropinosomes in Phafin2 knockout cells form transient tubular structures during their collapse (related to Figure 5). Images were acquired every 5s.

File Name: Supplementary Movie 13
Description: Phafin2 localizes to macropinosomes after scission (related to Figure 5). Cells expressing surface-displayed pH sensitive RFP (pHuji) and Phafin2-GFP were perfused with pH 5.5 and 7.5, leading to quenching and unquenching of pHuji fluorescence on exposed membranes, whereas sealed membranes are protected from pH changed. The first phase of Phafin2 localization occurs after the sealing of the vesicles. Images were acquired every 5s.

File Name: Supplementary Movie 14
Description: Light sheet movie of RPE1 cells expressing Phafin2-GFP and Lifeact-SNAP (related to Figure 6). Volumes were acquired every 10s.

File Name: Supplementary Movie 15
Description: Phafin2 and actin transiently colocalize on newly formed macropinosomes (related to Figure 6). Images were acquired every 5s.

File Name: Supplementary Movie 16
Description: Newly formed macropinosomes are surrounded by actin and are subjected to actin-based forces related to Figure 6). Macropinosomes are squeezed by actin forces and enter the cells through gaps in the actin network. Images were acquired every 5s.

File Name: Supplementary Movie 17
Description: Newly formed macropinosomes enter the cell through gaps in the surrounding FilaminA network (related to Figure 6). Images were acquired every 5s.

File Name: Supplementary Movie 18
Description: Effect of Phafin2 knockout on Filamin A recruitment (related to Supplementary Figure S6). Wild-type cells show transient FilaminA localization to newly formed macropinosomes; FilaminA

is shed from macropinosomes during their maturation. Macropinosomes in Phafin2 KO cells show prolonged FilaminA localization and are unable to shed FilaminA. This coincides with frequent collapse of the vesicle. Images were acquired every 5s.

File Name: Supplementary Movie 19
Description: Effect of Phafin2ΔC overexpression in cell morphology. Cells overexpressing Phafin2ΔC show increased cell retraction and membrane blebbing, and flat, filopodia-rich protrusions. Images were acquired every 5s.

File Name: Supplementary Movie 20
Description: High overexpression of Phafin2 induces massive membrane blebbing and amoeboid-like cell motility. Images were acquired every 5s.

File Name: Supplementary Movie 21
Description: Addition of the PI4K inhibitor GSK-A1 displaces Phafin2ΔC from the plasma membrane , rescues the cell retraction and membrane blebbing and restores normal cell shape. Images were acquired every 15s.

File Name: Supplementary Movie 22
Description: Light-induced plasma membrane recruitment of Phafin2 triggers cell retraction and membrane blebbing. Images were acquired every 5s.

File Name: Supplementary Movie 23
Description: Rapamycin-induced plasma membrane recruitment of Phafin2 triggers cell retraction and membrane blebbing. Images were acquired every 5s.

File Name: Supplementary Movie 24
Description: Light-induced plasma membrane recruitment of Phafin2 triggers cell retraction and membrane blebbing. Membrane blebs show transient de-lamination of the plasma membrane from the actin cytoskeleton. Images were acquired every 5s.
